# The combinatorial deletion of mycobacterial dd-carboxypeptidases is readily tolerated in Mycobacterium smegmatis

**DOI:** 10.1099/acmi.0.001074.v4

**Published:** 2025-12-19

**Authors:** Christopher S. Ealand, Danishka Moodley, Zaahida Sheik Ismail, Masethabela Maphatsoe, Lisa Campbell, Olivia Jacobs, Bavesh D. Kana

**Affiliations:** 1 DSI/NRF Centre of Excellence for Biomedical TB Research, School of Pathology, Faculty of Health Sciences, University of the Witwatersrand and the National Health Laboratory Service, Johannesburg, 2000, South Africa; 2 Infectious Diseases and Oncology Research Institute (IDORI), Faculty of Health Sciences, University of the Witwatersrand, Johannesburg, South Africa; 3 Wits Donald Gordon Medical Research Institute, Faculty of Health Sciences, University of Witwatersrand, Johannesburg, 2000, South Africa

**Keywords:** bacterial cell wall, dd-carboxypeptidase, *Mycobacteria*, peptidoglycan

## Abstract

Proteins facilitating bacterial cell wall (CW) biosynthesis are crucial for survival and broadly remain the target of numerous antimicrobial agents. Herein, we focused on characterizing the physiological roles of low-molecular-weight penicillin-binding proteins (LMW PBPs), with dd-carboxypeptidase (dd-CPase) activity, in *Mycobacterium smegmatis*. Following various combinatorial gene deletions, cell viability, colony structure and the ability to produce biofilms remained unperturbed. Whilst small changes in cellular morphology and permeability were evident, hierarchical roles could not be ascribed to specific dd-CPase homologues. Strains exposed to lysozyme exhibited low levels of compensatory expression for the remaining homologues, but this was not evident for exposure to the CW-targeting Augmentin. When tested against a broader concentration range of various antibiotics, using MIC and spotting assays, only marginal changes in drug susceptibility were evident. Strains cultured under conditions of excess NaCl or enhanced pH levels grew normally. Given the established role of remodelling in dd-CPase enzymes of other bacteria, we further assessed whether the ability to repair lysozyme-induced CW damage was compromised. With the incorporation of the fluorescent d-amino acid peptidoglycan probe, TAMRA-d-alanine, as a proxy for remodelling, no changes in staining patterns were evident. However, the frequency of cells containing unresolved septa increased in all mutant strains, suggesting a potential role for dd-CPases in the mycobacterial cell process. In conclusion, we have demonstrated that the combinatorial deletion of non-essential mycobacterial dd-CPase homologues largely has no significant impact on mycobacterial physiology or involvement in the response to the various environmental stressors tested herein.

## Data Summary

All data presented in this study are either included in the main article or in the supplementary information files. Further inquiries can be directed to the corresponding author.

## Introduction

The mycobacterial cell wall (CW) has historically provided a rich source of drug targets [1]. Of the four current first-line prophylactics to treat drug-sensitive tuberculosis (TB), isoniazid and ethambutol specifically inhibit mycolic acid and peptidoglycan (PG) biosynthesis, respectively, in the CW of the *Mycobacterium tuberculosis* (*Mtb*) [2,4]. The rapid emergence of drug-resistant strains has necessitated a constant need for the development of alternative chemotherapies. Whilst the *β*-lactam class of antibiotics has been used routinely to successfully treat various bacterial infections via the blocking of enzymes in the biosynthesis of the peptidoglycan component [5], mycobacteria are inherently resistant. This phenomenon is due to a thick, impermeable CW; the alteration of penicillin-binding proteins (PBPs) and potent genetically encoded *β*-lactamases [6,7]. PBPs, which are ubiquitous in all PG-containing bacterial species, are classified according to size, namely high or low molecular weight. Several chromosomally encoded homologues for both types are typically present in most bacterial genomes and their functions are associated with the maintenance of cellular morphology and/or PG biosynthesis [8,11]. These PBPs are predominantly acyl-serine transferases with either mono- (class B) or bi-functional (class A) catalytic activities – transpeptidases, transglycosylases, dd-carboxypeptidases (dd-CPases), ld-carboxypeptidases or endopeptidases [11,12]. Mycobacterial high-molecular-weight (HMW) PBPs have reported roles in growth dynamics, cellular morphology, survival during infection and susceptibility to antibiotics [13,19], whilst the contribution of low-molecular-weight penicillin-binding proteins (LMW PBPs) remains elusive. Despite this, and due to the genetic redundancy of mycobacterial LMW PBPs [20], it is likely that specialist roles emerge under specific environmental conditions. For example, together with PBP1b, DacA (PBP5) and DacC (PBP6) from *Escherichia coli* are required for growth under alkaline conditions, whereas only DacA is essential for growth during salt stress [21]. Deletion of DacA is also associated with an increased sensitivity to *β*-lactams and vancomycin resistance in *E. coli* and *Bacillus subtilis* [22], and in *B. subtilis* specifically, LMW PBP activity impacts endospore formation and PG remodelling thereof [23,25].

We have previously shown that *Mycobacterium smegmatis* (*Msm*) encodes four dd-CPase homologues [namely, MSMEG_1661, MSMEG_2432, MSMEG_2433 and MSMEG_6113 (*dacB*)], all of which are expressed during *in vitro* growth [20]. Individual deletion of three homologues separately resulted in no adverse physiological effects on growth, morphology, antibiotic susceptibility or spatial incorporation of new PG [26]. Repression of the essential homologue, *dacB*, resulted in altered PG biosynthesis patterns and remained paradoxical [26]. Other studies confirm that heterologous expression of MSMEG_2432 and MSMEG_2433 in *E. coli* PBP mutants restores aberrant cellular morphologies and increases *β*-lactam resistance, suggesting that mycobacterial homologues possess similar function [6,27]. In an *Msm* mutant strain lacking all ld-transpeptidases (∆*ldtABECFG*), severe morphology deformations were noted. Repression of MSMEG_2433 in this background reduced these abnormal phenotypes, pointing to a role in PG stabilization [28]. Augmentin (amoxicillin plus *β*-lactamase inhibitor) exposure altered the expression of all three *Mtb* dd-CPase homologues via the redox-responsive transcription factor, WhiB4, suggesting that dd-CPase activity is required for adaptation and/or PG remodelling [29]. In this context, the specific physiological roles of mycobacterial LMW PBPs remain incomplete. Herein, we build on our previous findings by creating various combinatorial dd-CPase deletion mutants in *Msm*. We further demonstrate that the simultaneous deletion of up to three dd-CPase genes minimally impacts mycobacterial physiology under various *in vitro* conditions.

## Methods

### Bacterial strains, media and culture conditions

The bacterial strains used in this study are listed in Table S1 (available in the online Supplementary Material). Unless otherwise indicated, *Msm* strains were grown in Middlebrook 7H9 media (Difco) supplemented with 0.2% glycerol, 1× glucose salts (final concentration of 0.2% glucose and 1.5 mM NaCl) and 0.05% Tween 80. Knockout vectors were propagated in *E. coli* using kanamycin and hygromycin (HYG) at a final concentration of 50 and 200 µg ml^−1^, respectively. Complemented derivatives in *Msm* were cultured in media supplemented with HYG at 50 µg ml^−1^.

### Allelic exchange mutagenesis

The plasmids used in this study are detailed in Table S1 and [26]. Essentially, all suicide plasmids used to introduce deletions (in-frame, unmarked) were constructed according to the protocols outlined in [30]. An additional plasmid, p2∆2432–2433 was constructed using the same approach, taking advantage of compatible sticky ends following restriction enzyme digests. This plasmid was used to simultaneously delete MSMEG_2432 and MSMEG_2433 from the bacterial chromosome. All knockout vectors were pre-treated by UV irradiation [31] and electroporated into the various single deletion mutant strains [26]. The p2∆2432–2433 vector was introduced directly into the wild-type strain, and to create the ∆triple mutant strain, p2∆1661 was electroporated into ∆2432_2433. Complementation vector p2∆1661 was electroporated into all mutant strains lacking MSMEG_1661, and integration into the *attB* site was confirmed by PCR.

### Colony morphology and establishment of liquid biofilms

To assess colony morphology defects, the respective bacterial strains were grown to mid-exponential phase (OD_600nm_~0.5–0.8) in 7H9 media (Middlebrook, Difco) supplemented with 1×glucose salts (GN) and 0.05% Tween 80. Ten microlitres of each was aliquoted onto solid 7H10 medium (i.e. 7H9+1.5% agar (w/v)) supplemented with 1×GN and allowed to air-dry. Plates were incubated at 37 °C for 3–4 days. Colony morphology images were captured using a digital camera (Nikon). To assess the pellicle biofilms formed at the liquid–air interface, all strains were incubated in 7H9 medium supplemented with 1×GN at 37 °C, with 100 r.p.m. shaking, until the stationary phase was reached. Cultures were then washed twice and adjusted to an OD_600nm_ of 1 in Sauton’s medium (pH 7.3). A 20 µl aliquot of each strain was added to 2 ml Sauton’s medium in 24-well culture plates (Nunc), sealed with Parafilm and incubated at 37 °C for 7 days. Biofilm images were captured using a digital camera (Nikon).

### Determination of cell dimensions

All strains were grown to mid-log phase (OD_600nm_~0.5–0.8), followed by 10 µl of each being spotted onto 1% agarose pads. Cell lengths and widths were determined by the manual measurement of at least 100 cells of three independent biological repeats (*N*=300). Images were captured on a Nikon Eclipse T12 and a PE4000 LED light source equipped with a Plan Fluor 100×oil immersion 1.30–numerical aperture objective. Images were captured in the TL DIC channel. The NIS-Elements AR software [Nikon Inc., Version 5.02 (Build 1266), Nikon Instruments Inc., Tokyo, Japan] was used to manually measure cell dimensions.

### Ethidium bromide diffusion assay to monitor cell permeability

*Msm* strains were grown in 20 ml of 7H9 medium at 37 °C to mid-log phase (OD_600nm_ ~0.5–0.8). Cultures were centrifuged at 3,901 ***g*** for 10 min, the supernatant discarded, and the pellet washed in PBST (1×PBS+0.1% Tween 80). Thereafter, the pellet was resuspended in PBSTG (PBST supplemented with 0.4% glucose) and the OD_600nm_ adjusted to 0.4. A 95 µl aliquot of the respective bacterial strains was placed into flat-bottom black 96-well plates (Corning^TM^). To a subset of the wells, carbonyl cyanide m-chlorophenyl hydrazone (CCCP) (Sigma-Aldrich) – an efflux pump inhibitor – was added to a final concentration of 50 µg ml^−1^ to serve as a positive control. The plate was then incubated at room temperature for 15–30 min before the addition of ethidium bromide (EtBr) to a final concentration of 8 µg ml^−1^. Fluorescence was measured every 60 s for 180 min at 37 °C (excitation and emission wavelengths of 530 nm and 585 nm, respectively). The accumulated levels of EtBr after 3 h were then compared across all strains. Three independent biological repeats, comprised of six technical repeats each, were set up for each strain±CCCP.

### Determination of MICs

For susceptibility assessments with antibiotics and lysozyme, axenic cultures for each strain were grown to mid-log phase (OD_600nm_ ~ 0.5–0.8) in 7H9 media. These were diluted 500-fold (OD_600nm_ = 0.001) in 7H9. Thereafter, a twofold dilution series was set up in a clear, 96-well, round-bottom plate as follows: 7H9 (50 µl) was aliquoted into each well, except for the top row of 8 wells. Antibiotics or lysozyme were added to the top row at a concentration in excess of the expected MIC in a final volume of 100 µl. Antibiotics were then diluted twofold from the top row (row 1) to the bottom row (row 12), starting with transferring 50 µl from row 1 to row 2 containing 50 µl 7H9. For the bottom row, 50 µl was discarded, so that all wells contained a final volume of 50 µl. After dilution of the antibiotics and controls (media only or vehicle only), 50 µl of the diluted cells was added to each well, bringing the final volume up to 100 µl. Plates were incubated at 37 ˚C and scored visually after 2 days, as growth or no growth. Following this, 10 µl of Alamar Blue (Thermo Fisher Scientific) was added to each well, and plates were further incubated at 37 °C for 3 h. Cell viability and subsequent determination of MIC were measured by scoring the colour change (reduction of resazurin from blue to pink). The MIC was recorded for the lowest concentration of drug at which no growth was observed.

### Quantitative real-time PCR and determination of compensatory gene expression

For exposure to lysozyme, 20 ml of each strain was grown to mid-log phase (OD_600nm_ ~ 0.5–0.8) in 7H9 media. This was then split equally and to half of the culture, lysozyme was added to a final concentration of 20 µg ml^−1^ (0.5×MIC). Cultures without lysozyme served as the negative control. After a further 6-h incubation (37 °C and shaking at 100 r.p.m.), the expression of each dd-CPase homologue was assessed by extracting RNA using the NucleoSpin^®^ RNA II Kit (Machery Nagel) as per the manufacturer’s instructions, following conversion to cDNA and real-time quantitative PCR analysis as previously described in [26]. For Augmentin exposure, compensatory gene expression was determined as described above except that lysozyme was replaced with amoxicillin (Sigma-Aldrich) (2 µg ml^−1^ or 0.25×MIC) and clavulanate (Sigma-Aldrich) (4 µg ml^−1^). All experiments were performed using three independent biological repeats. A *P-*value <0.05 was considered statistically significant, and this was determined using a two-sample t-test assuming unequal variances (Microsoft^®^ Excel^®^ for Microsoft 365 MSO; Version 2505 Build 16.0.18827.20102; 64-bit). For exposure to higher concentrations of either lysozyme (58.5 µg ml^−1^ or ~1.5× MIC) or Augmentin (12 µg ml^−1^ or ~1.5× MIC), experiments were set up as described above, but sampling times were reduced to 30 and 90 min.

### Spotting assays to determine susceptibility to antibiotics, enhanced pH or salt concentrations

All strains were grown to mid-log phase (OD_600nm_~0.5–0.8) in 7H9 media. A dilution series (10^0^–10^−4^) was prepared and 5 µl spotted onto 7H10 media either supplemented with 31.25 µg ml^−1^ amoxicillin, Augmentin (3.91 µg ml^−1^ amoxicillin+4 µg ml^−1^ clavulanate), increasing pH levels (pH 8–9.4) or increasing concentrations of NaCl (0.5–0.75 M). 7H10 plates without additional supplementation or pH adjustments served as a positive control for growth and viability. After allowing the aliquots to fully absorb into the media, plates were incubated at 37 °C for 3–4 days, followed by visual scoring.

### Lysozyme exposure and PG remodelling using TADA fluorescent PG probe

All strains were grown to mid-log phase (OD_600nm_ ~0.5–0.8) in 7H9 media. Two 500 µl aliquots of each bacterial culture were assigned to a±lysozyme condition (final concentration of 0 or 50 µg ml^−1^, respectively). Cell suspensions were incubated for a further 30 min (37 °C with shaking at 100 r.p.m.), after which 2.5 µl of TAMRA-d-Alanine (TADA) (final concentration of 5 µg ml^−1^) was added and incubated for a further 30 min (as above). Cell suspensions were then centrifuged at 13,000 r.p.m. for 5 min, and the supernatant was discarded. Bacterial pellets were washed in PBST (1×PBS+0.1% Tween 80) and centrifuged as above. Following the discarding of the supernatant, bacterial pellets were re-suspended in 250 µl PBST. A 10 µl aliquot of each strain (under the conditions of ±lysozyme with TADA) was spotted onto a 1% agarose pad. Images were visualized using a Nikon Ti2.0 fluorescence microscope [Channel 1=TL DIC or bright-field; Channel 2=TADA (maximum λ ex/em=554/580 nm, respectively) [32]]. Staining patterns were captured and scored using the NIS-Elements AR software (Nikon Inc., version 5.02, (Build 1266), Nikon Instruments Inc., Tokyo, Japan). All image manipulations, specifically brightness or contrast adjustments, were applied equally to the entire image. The staining patterns of at least 300 cells per condition, derived from three independent biological repeats (100 cells per repeat), were scored according to pre-defined staining patterns reflective of mycobacterial growth patterns.

## Results

### Combinatorial deletion of dd-CPases does not significantly alter cell morphology, viability, biofilm formation or cell wall permeability under standard laboratory conditions

**Fig. 1.**
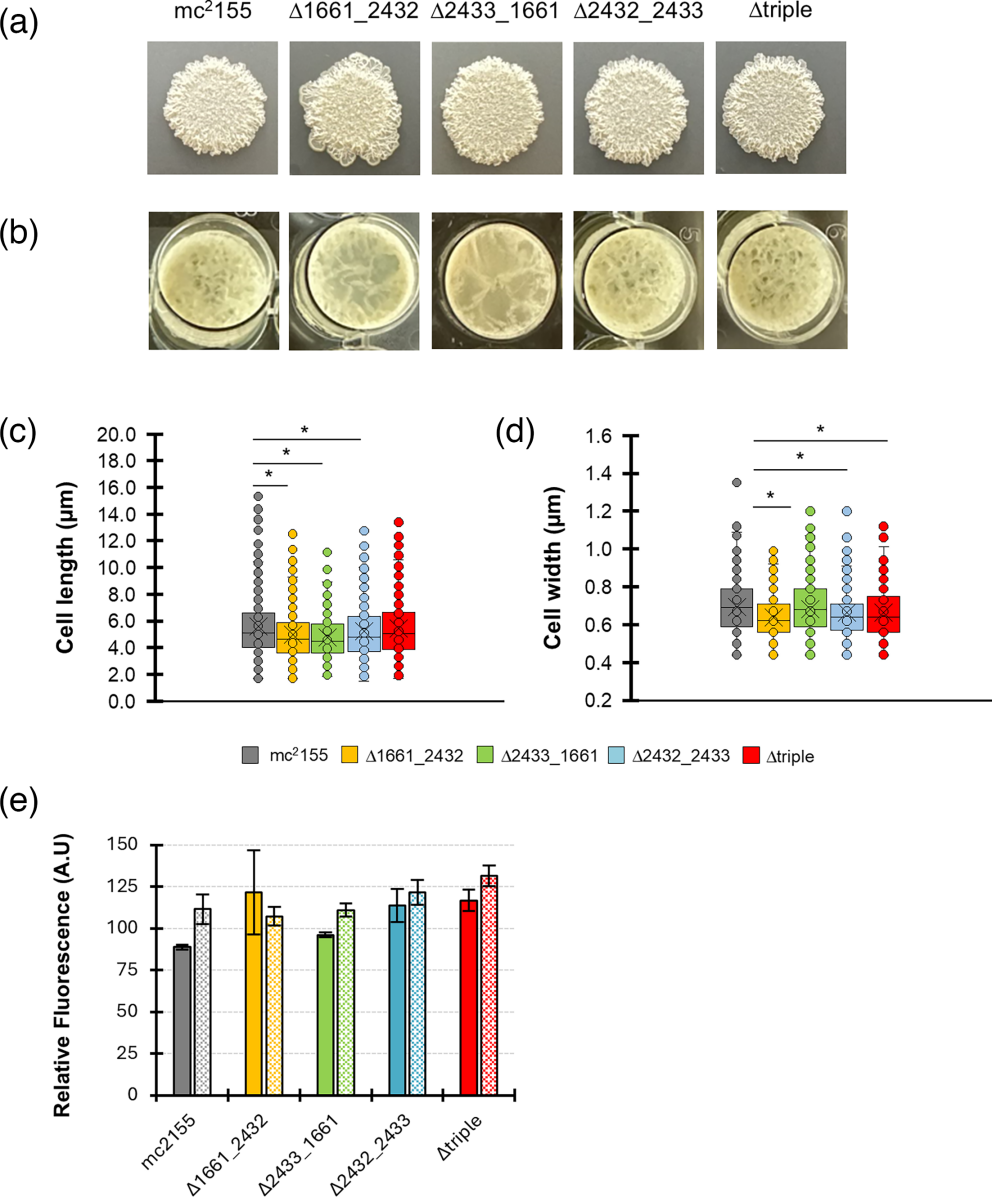
Effect of gene deletion on colony morphology, biofilm formation, cell dimensions and cell wall permeability. (**a**) *Msm* strains were grown to an optical density (OD_600nm_) of 0.5. A 10 µl aliquot was spotted onto solid media (7H10 supplemented with 1× glucose salts) and incubated at 37 °C for 3–4 days. Images of colonies were captured (1× magnification) using a digital camera. (**b**) Biofilm formation for *Msm* strains. All strains were adjusted to an OD_600nm_ of 1 and diluted 100-fold in Sauton’s minimal medium (pH 7.3) in 24-well plates. Plates were sealed with Parafilm and incubated at 37 °C without agitation for 7 days. Biofilms were then photographed using a digital camera (1× magnification) with images shown representative of three independent biological replicates. (**c and d**) Cell lengths and widths were determined by measuring a minimum of 300 cells (100 per biological repeat) at 100× magnification, using bright-field microscopy. A one-way ANOVA (Tukey’s multiple comparisons test) was used to compare means where statistically significant differences were shown (* represents a *P*<0.05). (**e**) Ethidium bromide diffusion assay. All strains in the exponential phase of growth (OD_600nm_ ~0.8) were washed and adjusted to 0.4 in PBSTG (1 x PBS+0.1% Tween 80+0.4% glucose). Ethidium bromide was added to a final concentration of 8 µg ml^−1^, and fluorescence levels were measured after 3 h at 530 and 585 nm (excitation and emission, respectively). The solid and hatched bars correspond to conditions without or with CCCP (50 µg ml^−1^), respectively. Data are representative of three independent experiments, each comprising six technical replicates and error bars indicating the standard error of the mean. A statistical analysis (two-way ANOVA) detected no statistically significant differences across or between groups (*P*≥0.05).

Similar to the genomes of *E. coli* and *B. subtilis*, we previously reported that mycobacterial species maintain a high level of redundancy for genes encoding PBPs [20]. Following the single deletions of LMW PBPs with putative dd-CPase activity in *Msm*, we reported no significant phenotypes related to cell morphology or growth rate *in vitro* [26]. As LMW PBP homologues have typically been implicated in the maintenance of cell morphology in other rod-shaped bacteria, we reasoned that adverse phenotypes might only manifest following combinatorial gene deletions in mycobacteria. To test this, we created a panel of mutants in *Msm*. Gene-specific deletions were all confirmed as correct using Southern hybridizations (Figure S1A–C). Relative to the wild-type strain (mc^2^155), mutants lacking double or triple gene deletion combinations displayed no adverse effects in colony morphology or biofilm formation. Moreover, all deletion mutant strains displayed similar growth rates compared to the wild type (Figure S2A, B). The typical cording phenotype associated with colonies grown on solid media and pellicle biofilms that form at the liquid-air interface both remained robust (Fig. 1a, b). Minor changes in cell length and cell width were evident, and whilst there were statistically significant differences in these dimensions, it was difficult to ascribe a role to individual dd-CPase homologues as the average length of the ∆triple mutant strain was not different from the wild-type strain (Fig. 1c, d). Owing to the marginal changes observed in cell lengths and widths, we speculated that this could contribute to alterations in CW permeability as reported in our previous studies for other PG-associated enzymes [33,34]. To test this in the dd-CPase-deficient mutant strains, permeability was assessed via an ethidium bromide diffusion assay. An additional control, CCCP (50 µg ml^−1^), was included with all strains to serve as an efflux pump inhibitor. In this case, ethidium bromide was expected to be maintained at higher levels. As observed in Fig. 1e, all the combinatorial deletion strains displayed similar levels of ethidium bromide accumulation, relative to the wild type, after 3 h. Moreover, in the presence of CCCP, ethidium bromide levels were consistently higher for each strain. Despite this, statistical testing detected no significant differences across or between groups (*P*>0.05 for all comparisons). The lack of significant macro-scale phenotypes following the combinatorial deletion of dd-CPase genes suggested that possible impacts on bacterial physiology were more nuanced and possibly mitigated by the presence of the essential gene, MSMEG_6113 (*dacB*), which cannot be deleted from the genome.

### Compensatory expression of *dacB* in the background of combinatorial dd-CPase deletions

**Fig. 2.**
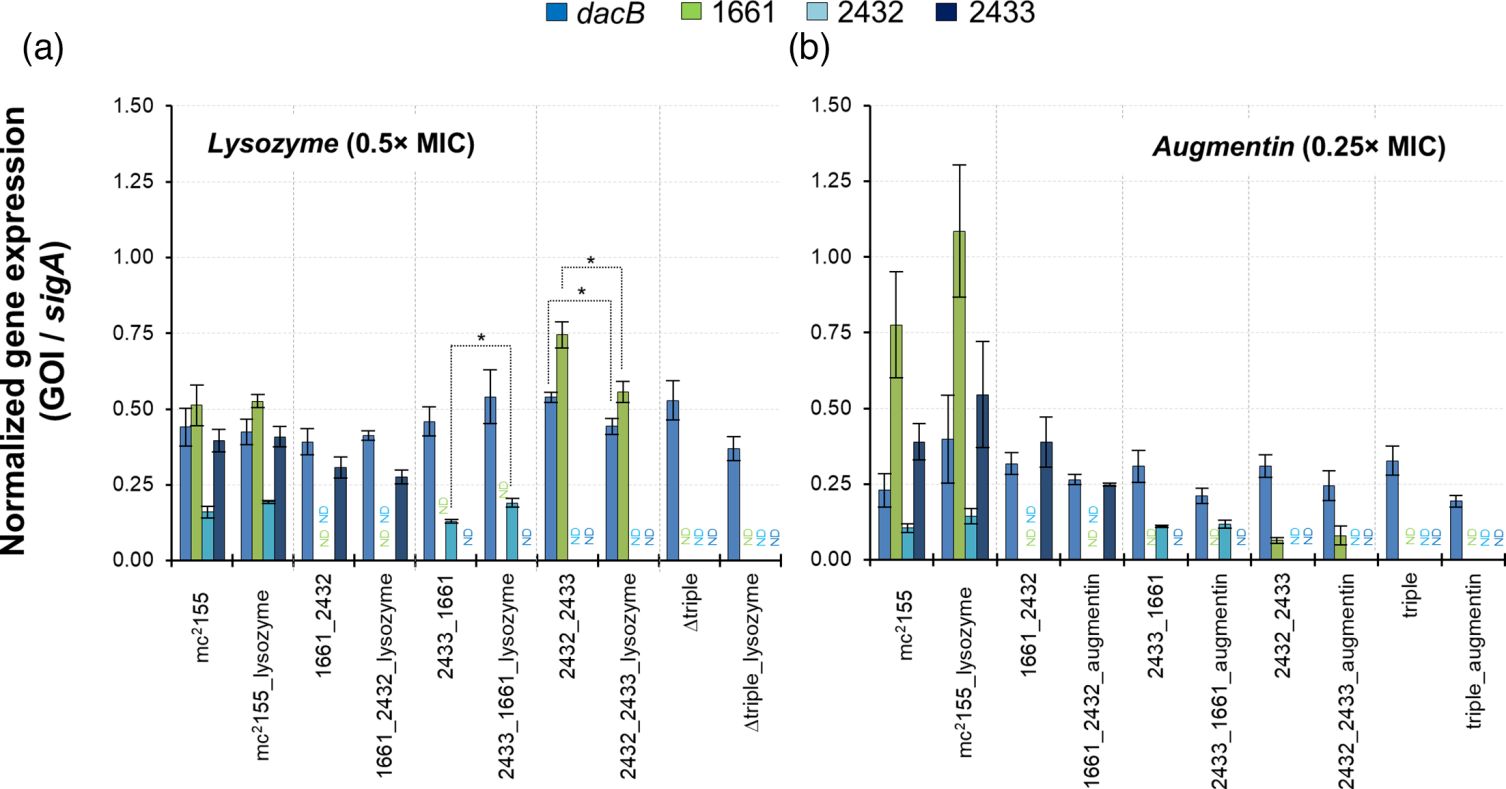
Determination of compensatory expression by remaining dd-CPase homologues in the respective combinatorial deletion mutant strains. (**a**) Exponentially growing strains (OD_600nm_ of 0.5–1.0) were exposed to lysozyme (0.5ˣ MIC at 20 µg ml^−1^). Cultures lacking lysozyme served as the negative control. After the incubation period (6 h at 37 °C with 100 rpm shaking), total RNA was extracted and converted to cDNA. All gene-specific dd-CPase transcripts were quantified using genomic DNA standard curves and normalized against the housekeeping gene, *sigA*. Failure to detect a transcript served as an additional control in the respective mutant strains and is indicated by ‘ND’ (not detected). (**b**) The expression of dd-CPase genes in strains exposed to Augmentin (0.25ˣ MIC at 2 µg ml^−1^ amoxicillin+4 µg ml^−1^ clavulanate) was determined as described above. No drug exposure served as the negative control. Under all experimental conditions, data are representative of three independent biological repeats. Error bars depict the standard error of the mean. *P*<0.05 (considered statistically significant) was determined using t-tests comparing the means of transcript levels in the presence or absence of lysozyme or Augmentin (two-sample assuming unequal variances). Only statistically significant differences are shown with an *.

In all four mutant strains tested in this study, *dacB* was still present. We previously showed that all four homologues were differentially expressed during early-log, mid-log, late-log and stationary growth phases [26]. We, therefore, postulated that in the absence of various combinations of dd-CPase deletions, compensatory expression of the remaining homologues could mitigate the broader loss of dd-CPase activity. To interrogate this further, we assessed dd-CPase expression in bacterial cells derived from exponentially growing cultures under standard laboratory conditions and when exposed to lysozyme (20 µg ml^−1^ or 0.5×MIC) or Augmentin (31.25 µg ml^−1^ amoxicillin+4 µg ml^−1^ clavulanate or 0.25×MIC) for 6 h. In either scenario, the mycobacterial CW was expected to be damaged via enzymatic degradation in the PG layer or by the blocking of PBPs responsible for PG biosynthesis, respectively. With respect to lysozyme, *dacB* was expressed at equal levels in the wild-type strain in the absence and presence of the CW-damaging agent (0.44 vs. 0.42, *P*>0.05, respectively). This response was similar for the *sigA*-normalized transcripts of MSMEG_1661, MSMEG_2432 and MSMEG_2433 (0.51 vs. 0.53 (*P*>0.05); 0.16 vs. 0.19 (*P*>0.05); and 0.40 vs. 0.41 (*P*>0.05), respectively) (Fig. 2a). In ∆1661_2432, transcripts for MSMEG_1661 and MSMEG_2432 were not detected as expected (Fig. 2a). However, the *dacB* transcript levels remained unchanged [0.39 vs. 0.41 (*P*>0.05)] in the absence or presence of lysozyme. A similar pattern was observed for MSMEG_2433 [0.31 vs. 0.28 (*P*>0.05), respectively]. In ∆2433_1661 (Fig. 2a), the *dacB* transcript levels increased from 0.46 (absence of lysozyme) to 0.54 (presence of lysozyme) (*P*>0.05), but this was not statistically significant. In contrast, the transcript levels for MSMEG_2432 displayed a marginal, but statistically significant increase (*P*<0.05) in the presence of lysozyme (0.13 vs. 0.19, respectively). In the ∆2432_2433 strain (Fig. 2a), *dacB* transcript levels dropped significantly from 0.54 in the absence of lysozyme to 0.44 in its presence (*P*<0.05). A similar statistically significant drop in the expression of MSMEG_1661 was observed [0.75 vs. 0.56 (*P*<0.05), respectively]. In the ∆triple strain (Fig. 2a), *dacB* transcript levels dropped from 0.53 to 0.37 in the absence or presence of lysozyme. Taken together, these data suggest that in the absence of other dd-CPase homologues, the remaining genes do respond and may sense and/or respond to PG damage.

With respect to Augmentin, *dacB* was expressed at low levels in cultures, derived from the wild-type strain, lacking Augmentin (0.23), but this increased following exposure to the drugs (0.40), albeit not at statistically significant levels (*P*>0.05) (Fig. 2b). Similarly, the transcript levels for MSMEG_1661, MSMEG_2432 and MSMEG_2433 increased in response to the drug (0.78 vs. 1.09, 0.10 vs. 0.14, and 0.39 vs. 0.55, respectively), but this was not statistically significant (*P*>0.05). In the ∆1661_2432 strain (Fig. 2b), both *dacB* and MSMEG_2433 transcript levels decreased (0.32 vs. 0.27 and 0.39 vs. 0.25, respectively), but this was not statistically significant (*P*>0.05, respectively). In the ∆2433_1661 strain (Fig. 2b), a similar decrease in *dacB* transcript levels (0.31 vs. 0.21) with no statistically significant changes (*P*>0.05). MSMEG_2432 transcript levels remained relatively constant [0.11 vs. 0.12 (*P*>0.05)]. In the ∆2432_2433 strain (Fig. 2b), *dacB* transcript levels dropped slightly in response to Augmentin [0.31 vs. 0.24 (*P*>0.05)], whereas MSMEG_1661 levels appeared to be consistently repressed [0.06 vs. 0.08 (*P*>0.05)]. Relative to the wild-type strain, MSMEG_1661 transcript levels were ~13-fold less in both conditions, suggesting that the simultaneous lack of MSMEG_2432 and MSMEG_2433 impacts MSMEG_1661 levels. In the ∆triple strain (Fig. 2b), *dacB* transcript levels dropped in response to Augmentin (0.33 vs. 0.19), but this was trending towards statistical significance (*P*=0.06). Taken together, despite the general lack of statistically significant changes in gene expression, these data trend towards decreased expression of the remaining dd-CPase encoding genes in response to Augmentin, suggesting that these proteins might be required for antibiotic-induced PG disruption. We next postulated that because changes in dd-CPase gene expression were observed, it was possible that more pronounced compensatory effects would be detected following exposure to higher concentrations of lysozyme (58.5 µg ml^−1^ or 1.5×MIC) or Augmentin (12 µg ml^−1^ or 1.5×MIC). To prevent the activation of cell death pathways, we adjusted the sampling to include two earlier time points (i.e. 30- or 90-min post-exposure). Overall, higher concentrations of lysozyme or Augmentin failed to significantly impact compensatory gene expression patterns (Fig. S3A–D).

### Sensitivity to CW-targeting agents is influenced by cellular dd-CPase activity but not alkaline or salt stress in *Msm*

We next reasoned that if differential regulation of dd-CPase encoding genes occurred in response to Augmentin in *Mtb* [29] and lysozyme (as reported for the dd-CPase transcriptional analyses herein), this could potentially point to a hierarchy of dd-CPase function in *Msm*. In *Mtb* specifically*,* genetic determinants of *β*-lactam tolerance include PG biosynthetic genes, cell division and DNA transcription factors, DNA repair genes and PBPs (including the LMW PBP *dacB*), which are mediated by the redox-sensitive transcription factor, WhiB4 [29]. Moreover, the same genes have previously been implicated with *β*-lactam tolerance in other bacterial species [35]. To further investigate this phenomenon in *Msm*, we determined the MICs for the CW-targeting agents: amoxicillin, ethambutol, lysozyme and vancomycin. Rifampicin served as a positive control as it is used as part of first-line therapy targeting *Mtb*.

**Fig. 3.**
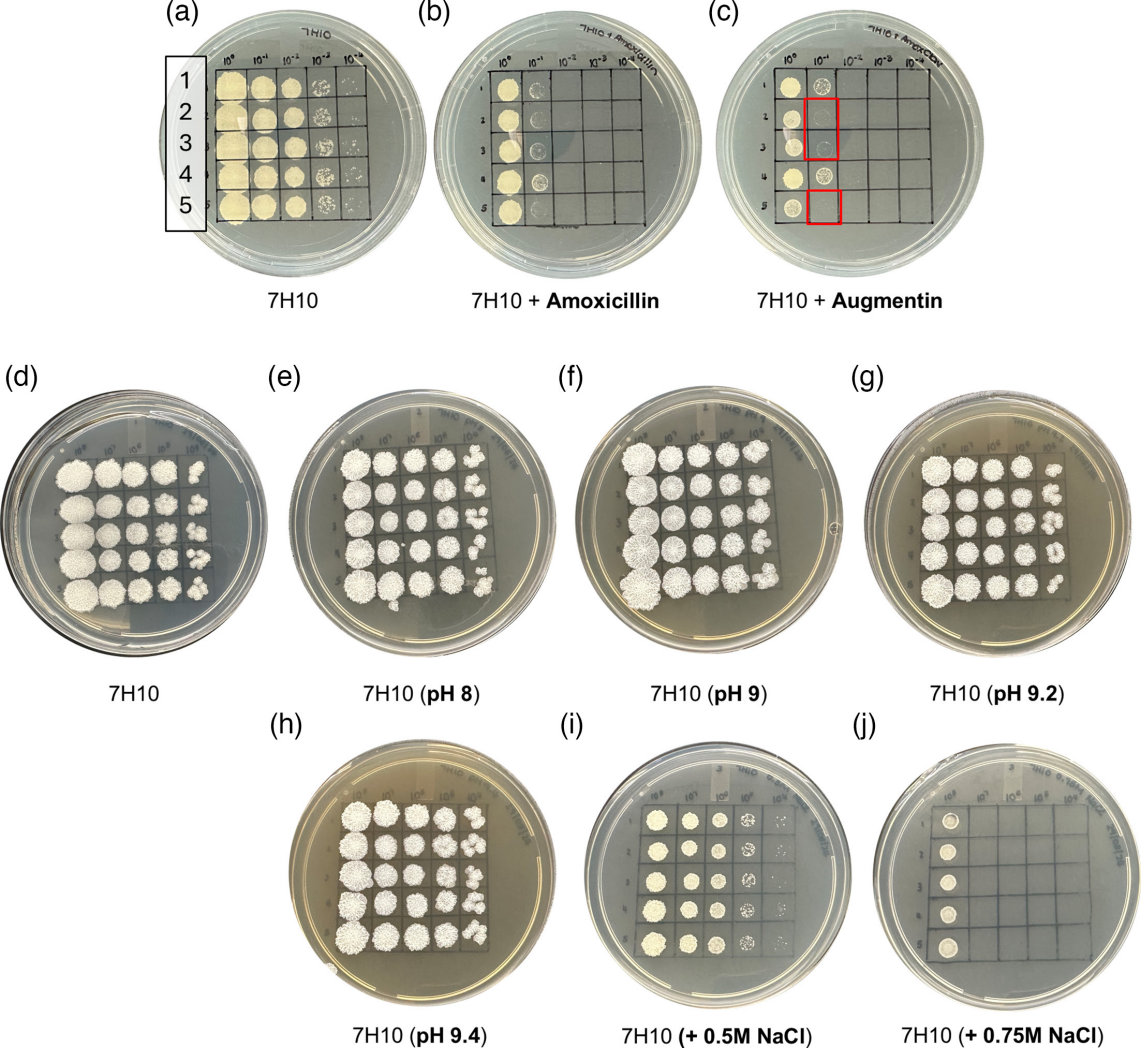
dd-CPase activity mediates the response to Augmentin in *Msm* but not alkaline or salt stress. (**a**) 7H10 only to serve as the control. (**b**) 7H10 supplemented with 31.25 µg ml^−1^ amoxicillin. (**c**) 7H10 supplemented with Augmentin (3.91 µg ml^−1^ amoxicillin+4 µg ml^−1^ clavulanate). In all conditions, exponential-phase cultures of each strain were serially diluted tenfold (1=mc^2^155; 2 = ∆1661_2432; 3 = ∆2433_1661; 4 = ∆2432_2433; and 5 = ∆triple) and 5 µl spotted onto media. Plates were then incubated at 37 °C for 3–4 days, followed by scoring. Red boxes indicate differential susceptibility to killing by Augmentin. (**d**) 7H10 only to serve as the control. (**e**) 7H10 adjusted to pH 8 with NaOH. (**f**) 7H10 adjusted to pH 9 with NaOH. (**g**) 7H10 adjusted to pH 9.2 with NaOH. (**h**) 7H10 adjusted to pH 9.4 with NaOH. (**i**) 7H10 supplemented with 0.5M NaCl. (**j**) 7H10 supplemented with 0.75M NaCl. Plates were prepared, incubated and scored as described above.

Overall, only marginal changes in the MICs of the antibiotics tested were observed for the combinatorial deletion mutant strains (Table 1). The MIC for amoxicillin ranged between 31.25 and 62.50 µg ml^−1^ for all strains. For all other compounds tested (i.e. ethambutol, lysozyme, rifampicin or vancomycin), no changes in susceptibility were observed. To interrogate Augmentin (amoxicillin combined with clavulanate) sensitivity more closely, log-phase cultures for each strain were spotted onto solid media supplemented with amoxicillin or Augmentin. In this case, all the mutant strains lacking MSMEG_1661 (i.e. ∆1661_2432, ∆2433_1661 and ∆triple) appeared to be tenfold more sensitive to killing by Augmentin (Fig. 3a–c). Complementation of these strains with MSMEG_1661 reversed this sensitivity, suggesting a potential hierarchy of dd-CPase function following exposure to Augmentin (Fig. S4).

**Table 1.** Susceptibility of dd-CPase deletion mutants to antibiotic and cell wall-damaging agents*

Compound	mc^2^155	∆1661_2432	∆2433_1661	∆2432_2433	∆triple
Amoxicillin	31.25–62.50	31.25	62.50	62.50	62.50
Ethambutol	0.31–0.63	0.31–0.63	0.31–0.63	0.31–0.63	0.31–0.63
Lysozyme	39.06	39.06	78.12	39.06–78.12	39.06
Rifampicin	0.39–0.78	0.3–0.78	0.39–0.78	0.39–0.78	0.39–0.78
Vancomycin	0.31–0.63	0.31–0.63	0.31–0.63	0.31–0.63	0.31–0.63

* MICs were determined using the broth microdilution method, consisting of two fold dilutions, and are reported in mg l−1. Data are representative of 3 independent biological repeats with 16 technical replicates each.

Distinct roles for the LMW PBPs DacA and DacC in cell growth and shape maintenance under alkaline or salt stress conditions have been previously reported in *E. coli* [21]. We, therefore, sought to establish whether dd-CPases played similar roles in *Msm*. Log-phase cultures for each strain were serially diluted and spotted onto 7H10 media either pH-adjusted (pH range=8–9.4) or supplemented with excess NaCl (0.5–0.75M NaCl). Standard 7H10 media served as the control media. The combinatorial deletion of up to three distinct dd-CPase homologues had no adverse effect on bacterial survival under increased alkaline or excess salt conditions relative to the wild-type strain (Fig. 3d–j).

### Remodelling of CW damage by lysozyme treatment

**Fig. 4.**
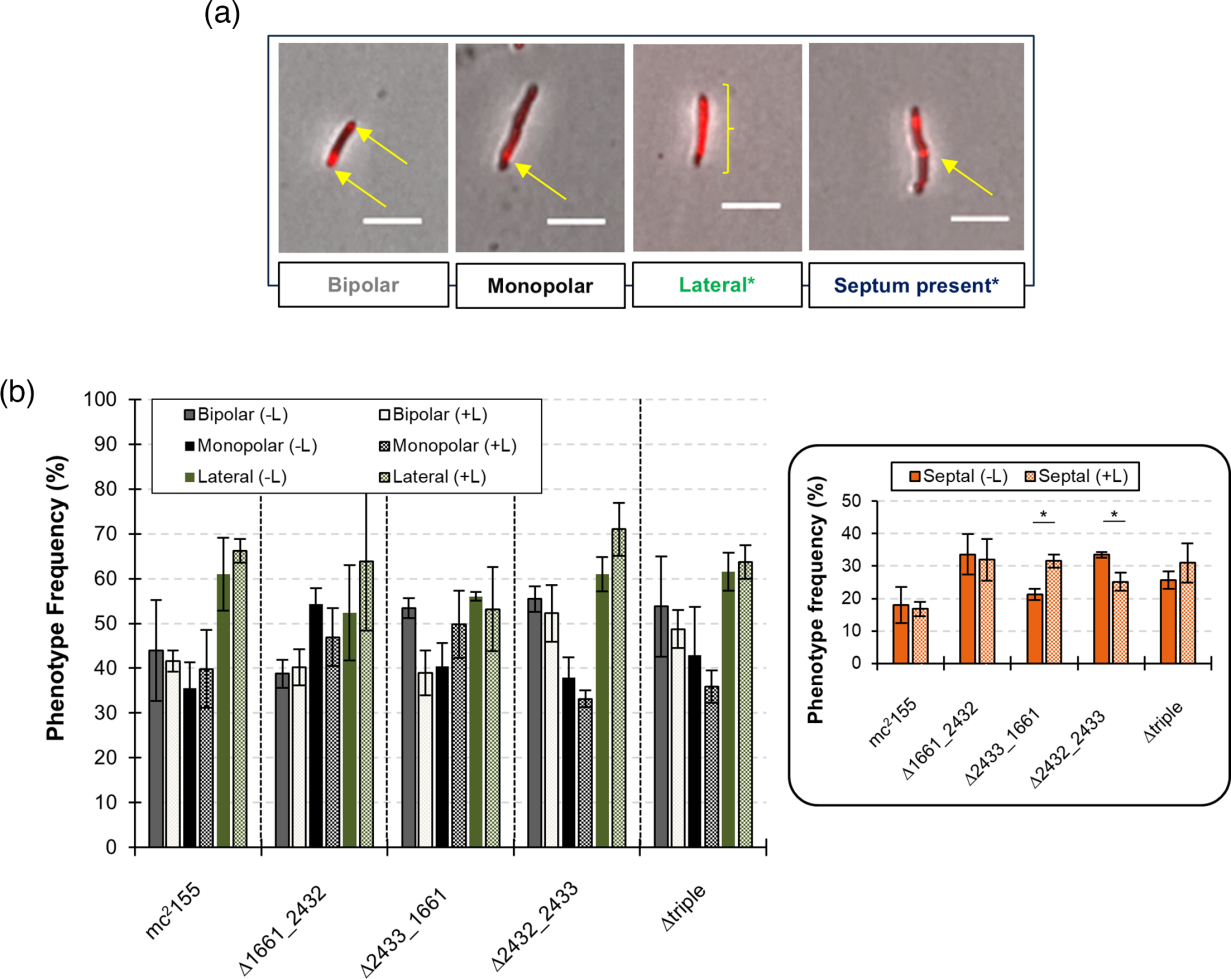
Role of dd-CPases in peptidoglycan remodelling in response to lysozyme degradation. (**a**) Representative images for staining patterns scored are indicated by yellow arrows or brackets. *Where enhanced lateral staining and/or the presence of a septum were noted, these could co-occur with bipolar or monopolar staining. Thus, one cell could hypothetically be scored as bipolar or monopolar with simultaneous lateral staining or the presence of a septum. Due to this scoring approach, the percentages reported may exceed 100%. (**b**) Phenotype frequency (%) of staining patterns observed in the absence (-L) or presence (+L) of lysozyme. Inset. Phenotype frequency (%) of cells containing septa. Data are represented as the means±standard error of the mean for three independent biological repeats (*n*=minimum of 300 cells counted for each strain). *P*<0.05 (considered statistically significant) was determined using t-tests comparing phenotype frequencies in the presence or absence of lysozyme (two-sample assuming equal variances). Only statistically significant differences are shown with an *.

We next postulated that because dd-CPase activity is associated with PG stem peptide remodelling, lysozyme-induced damage and subsequent repair would be altered in mutants with diminished dd-CPase activity. To investigate this further, we used a combination of lysozyme treatment followed by staining with the fluorescent d-amino acid PG probe, TADA [32]. Cultures lacking lysozyme served as the CW damage-free control. Probe uptake, as a proxy for PG remodelling, was scored as ‘bipolar’, ‘monopolar’, ‘lateral’ and/or ‘septum present’. It was worth noting that septa or enhanced lateral staining could also be present in either of the polar-type classifications above (Figs 4a and S6). As such, the cumulative frequencies of staining patterns observed in cells could exceed 100%.

In the wild-type strain (Fig. 4b), lysozyme did not impact the canonical growth pattern of mycobacteria [36] as ‘bipolar’ staining was evident in most cells in the absence (~44%) or presence of lysozyme (~42%) (*P*>0.05). Approximately 35% of cells displayed a ‘monopolar’ staining pattern and when lysozyme was added to the culture, a marginal increase (~40%) was evident (*P*>0.05). A large proportion (~60%) of ‘lateral staining’ was evident, but this was not significantly increased (~66%) in cultures containing lysozyme (*P*>0.05). A septum was present in ~18% of cells, but lysozyme failed to negatively impact cell division with only a minor decrease in septum-containing cells (~16%) (*P*>0.05). In the ∆1661_2342 strain (Fig. 4b, inset), the ‘bipolar’ staining pattern was comparable in the presence and absence of lysozyme [~39 and 40%, respectively (*P*>0.05)]. The ‘monopolar’ staining pattern was observed in ~54% of cells, but this did not decrease significantly (~47%) following exposure to lysozyme (*P*>0.05). ‘Lateral staining’ was evident in ~52% of cells, and this increased to ~64% with lysozyme treatment (*P*>0.05). Septa were present in ~34% of cells not exposed to lysozyme, but this decreased marginally (~32%) in the presence of lysozyme (*P*>0.05). In the ∆2433_1661 strain (Fig. 4b, inset), ~53% of cells adopted a ‘bipolar’ staining pattern. Upon exposure to lysozyme, this dropped to ~39%, trending towards statistical significance (*P*>0.05). Concomitantly, the frequency of ‘monopolar’ staining appeared to increase accordingly following exposure to lysozyme because in cells not exposed to lysozyme, ~40% of cells adopted the ‘monopolar’ staining pattern. The addition of lysozyme caused this increase to ~50% (*P*>0.05). Almost equal proportions of cells displaying ‘lateral staining’ were evident with or without lysozyme [53 and 56% (*P*>0.05), respectively]. Septa were present in ~21% of cells in the absence of lysozyme, but this increased to ~32% following its inclusion, which appeared to be statistically significant (*P*<0.05). In the ∆2432_2433 strain (Fig. 4b, inset), ~56% and 53% of cells displayed the ‘bipolar’ staining pattern in the absence and presence of lysozyme, respectively (*P*>0.05). This was associated with a lower frequency of the ‘monopolar’ staining pattern under the same conditions at ~38 and ~33%, respectively (*P*>0.05). ‘Lateral staining’ increased from ~61% in cells not exposed to lysozyme to ~71% in the presence of it (*P*>0.05). The frequencies of cells containing ‘septa’ in cultures lacking or containing lysozyme showed a statistically significant decrease (~34 vs. ~25%, respectively) (*P*=0.05) (Fig. 4b, inset). In ∆triple, staining patterns for ‘bipolar’, ‘monopolar’, ‘lateral staining’ and ‘septum present’ were comparable in the absence and presence of lysozyme with no statistically significant differences observed (Fig. 4b). Interestingly, in all the combinatorial deletion mutant strains, the presence of a septum was increased by ~2-fold (*P*>0.05) relative to the wild-type strain regardless of lysozyme treatment (Fig. 4b, inset), suggesting that the simultaneous deletion of MSMEG_1661, MSMEG_2432 and MSMEG_2433 possibly influences the rate of cell division in *Msm*.

## Discussion

The biosynthesis and remodelling of various components in the bacterial cell walls have long been the target of various antibiotics [37,40]. Of particular concern is that bacterial species of the genus *Mycobacterium*, including notable human pathogens from the *Mycobacterium tuberculosis* complex, *Mycobacterium avium* and the *Mycobacterium abscessus* complex, which continue to contribute to the high burden of pulmonary/disseminated disease and/or skin infections in countries with a high prevalence of Human Immunodeficiency Virus /AIDS and TB. Moreover, mycobacterial species are inherently tolerant/resistant to conventional antibiotics due to a complex cell wall and a multiplicity of efficient compound-degrading enzymes [41,42]. With the rapid emergence of antimicrobial resistance, even well-established, conventional therapies are becoming ineffective.

Whilst the role of PBP5 (or related homologues) in other bacteria with respect to cell morphology and PG modifications is well established [12,18, 19, 42,46], our previous study suggested that single gene deletions were easily tolerated with minimal adverse effects on mycobacterial physiology [26], corroborating the phenotypes observed for the single gene deletions of MSMEG_2432 and MSMEG_2433 reported in [47]. Heterologous expression of either MSMEG_2432 or MSMEG_2433 in the septuple PBP mutant of *E. coli* restored cell morphology defects, highlighting its functional role in PG biology [27,48]. In contrast, the single deletion of DacA-1, a PBP5 homologue, in *Vibrio cholerae* was associated with slow growth, abnormal morphology, a plating defect and altered PG homeostasis. Despite genetic multiplicity, DacA-1 alone caused these growth defects as mutants lacking DacA-2 and/or homologues of PBP4/7 grew normally [49].

Herein, we created a panel of combinatorial dd-CPase gene deletions in *Msm* to further investigate the interplay between the various homologues and environmental stressors. At the macroscopic level, all deletion mutant strains displayed no altered colony morphologies, compromised liquid biofilm forming capability, changes in growth rate (Fig. S2) or CW permeability. At the microscopic level, combinatorial gene deletions were associated with minor changes in cell length and width, but these phenotypic differences were challenging to reconcile due to the triple mutant’s morphology being comparable to the wild-type strain. Pandey and colleagues [47] previously reported that the simultaneous deletion of MSMEG_2432 and MSMEG_2433 was associated with altered cross-link formations, a reduction in cell surface glycopeptidolipid expression and differential susceptibility to antimicrobial agents, suggesting that dd-CPase activity significantly influences CW integrity. Whilst we did not test the cell surface modifications in our equivalent strain (∆2432_2433), we noted that all combinatorial deletion mutants were marginally more susceptible to Augmentin but not amoxicillin, ethambutol, rifampicin, vancomycin or lysozyme. In *E. coli*, deletion of PBP5 was associated with increased susceptibility to all the *β*-lactam antibiotics tested [50]. Interestingly, heterologous complementation with PBP5 homologues from *Salmonella enterica*, *Vibrio cholerae* and *Haemophilus influenzae* all restored, either completely or partially, the lost *β*-lactam resistance [50], suggesting a direct role of these enzymes in antibiotic tolerance. As PBP5 aids in maintaining *β*-lactam resistance within the *E. coli* cell, Sarkar *et al.* interrogated the roles of PBP6 and DacD by deleting these individually and in combination with DacA [PBP5] [51]. PBP5 dd-CPase activity was shown to be essential for intrinsic *β*-lactam resistance, whereas DacD could partially compensate for the loss of PBP5 and PBP6 could not [51]. In our mutant strains lacking MSMEG_1661, populations displayed a log-fold difference in survival when plated on Augmentin, thus supporting a similar role for these enzymes in mycobacteria. In *Pseudomonas aeruginosa,* LMW PBP activity is associated with *β*-lactam resistance, which is likely regulated by the interplay between their roles in AmpC induction, *β*-lactam trapping and dd-CPase/*β*-lactamase activities [52].

As reported in our previous study, a sole dd-CPase homologue, MSMEG_6113 (*dacB*), was essential and could not be deleted from the genome of *Msm*. Repression of *dacB* and the downstream genes in the operon consequently resulted in altered PG biosynthesis [26]. In the context of this present study, *dacB* was still present in the deletion mutant strains constructed. We reasoned that in the absence of certain combinations of dd-CPases, expression of *dacB* would be influenced, as a compensatory response, in the presence of antimicrobial agents. Interestingly, all the dispensable dd-CPase homologues contain signal peptides directing these proteins to the periplasm, whereas *dacB* does not, suggesting that its function is confined to the cytoplasm (Fig. S5). It is, therefore, plausible that the periplasmic proteins serve to remodel PG and/or sense PG fragments due to mechanical or antibiotic-induced damage. Transcriptional regulation of select homologues was evident in the presence of lysozyme, but not Augmentin, suggesting possible sensing in response to PG damage.

Collectively, this study has demonstrated that the combinatorial deletion of up to three dd-CPases in *Msm* had no significant negative impact on viability, growth, CW permeability or antibiotic susceptibility, unlike the dd-CPase homologues present in more canonical rod-shaped bacteria. A comprehensive chemical analysis describing the PG ultra-structure would elucidate the chemical modifications effected by these mycobacterial enzymes more clearly, forming the basis of future studies. By understanding how dd-CPases function together with proteins in the mycobacterial divisome and/or elongasome, novel vulnerabilities could be identified and exploited for rational drug design in mycobacterial pathogens.

## Supplementary material

10.1099/acmi.0.001074.v4 Uncited Fig. S1.

## Abbreviations

CCCP: carbonyl cyanide m-chlorophenyl hydrazone
CW: cell wall
dd-CPase: dd-carboxypeptidase
HMW: high-molecular-weight
HYG: hygromycin
LMW PBPs: low-molecular-weight penicillin-binding proteins
*Msm*: *Mycobacterium smegmatis*
*Mtb*: *Mycobacterium tuberculosis*
PBPs: penicillin-binding proteins
PG: peptidoglycan
TADA: TAMRA-d-alanine
TB: tuberculosis
