# Supplementary material for: The combinatorial deletion of mycobacterial dd-carboxypeptidases is readily tolerated in Mycobacterium smegmatis
Source: Access Microbiol. 2025 Dec 19;7(12):001074.v4. doi: 10.1099/acmi.0.001074.v4 (PMC12795238; doi:10.1099/acmi.0.001074.v4)
Supplement: Uncited Fig. S1. [file acmi-7-01074-s001.pdf]

The combinatorial deletion of mycobacterial DD-carboxypeptidases is readily tolerated in *Mycobacterium smegmatis*

SUPPLEMENTARY INFORMATION

Christopher Ealand[1#], Danishka Moodley[1], Zaahida Sheik Ismail[1,2], Masethabela Maphatsoe[1,3], Lisa Campbell[1,4], Olivia Jacobs[1,5] and Bavesh Kana[1*#]

Affiliations:

[1]DSTI/NRF Centre of Excellence for Biomedical TB Research, School of Pathology, Faculty of Health Sciences, University of the Witwatersrand and the National Health Laboratory Service, Johannesburg, 2000, South Africa.

[2]Current address: Holistic Drug Development and Discovery Center (H3D), University of Cape Town (UCT), Cape Town, 7701, South Africa.

[3]Current address: Industrial Microbiology & Biotechnology Laboratory, School of Molecular and Cell Biology, University of the Witwatersrand, 2000 Johannesburg, South Africa.

[4]Wits Donald Gordon Medical Research Institute, Faculty of Health Sciences, University of Witwatersrand, Johannesburg, 2000, South Africa.

[5]Current address: The Institute of Biochemistry, Johann Wolfgang Goethe Universit, Frankfurt am Main, 60439, Germany.

[#]Infectious Diseases and Oncology Research Institute (IDORI), Faculty of Health Sciences, University of the Witwatersrand, Johannesburg, South Africa.

Key words: Mycobacteria; Bacterial cell wall; Peptidoglycan; DD-carboxypeptidase.

Corresponding author: Mailing address: DSI/NRF Centre of Excellence for Biomedical TB Research, National Health Laboratory Service, P.O. Box 1038, Johannesburg, 2000, South Africa. Phone: Tel: +(27)114899135; Fax: +(27)114899397; Email: Bavesh.Kana@wits.ac.za

Supplementary Tables

Table S1. Strains and plasmids used in this study.

Strains	Description	Source/ Reference
mc^{2}155	Efficient plasmid transformation mutant of mc26	(1)
Δ1661_2432	Derivative of ΔMSMEG_1661 carrying an unmarked, in-frame deletion in *M. smegmatis* MSMEG_1661 (1158 bp internal region excised) in addition to MSMEG_2432 (786 bp internal region excised).	This study
Δ2433_1661	Derivative of ΔMSMEG_2433 carrying an unmarked, in-frame deletion in *M. smegmatis* MSMEG_2433 (870 bp internal region excised) in addition to MSMEG_1661 (786 bp internal region excised).	This study
Δ2433_2432	Derivative of mc^{2}155 carrying an unmarked, in-frame deletion of MSMEG_2432 (786 bp internal region excised), MSMEG_2433 (870 bp internal region excised) and the intergenic region between the two genes.	This study
Δ1661_2432_2433 (Δtriple)	Derivative of Δ2433_2432 carrying an unmarked, in-frame deletion of MSMEG_1661 (786 bp internal region excised).	This study
Δ1661_2432::1661	Derivative of ΔMSMEG_1661 carrying an unmarked, in-frame deletion in *M. smegmatis* MSMEG_1661 (1158 bp internal region excised) in addition to MSMEG_2432 (786 bp internal region excised). Complementation vector pMV306H_1661 carrying the entire MSMEG_1661 gene and 150bp upstream region inserted into the bacterial *attB* site.	This study
Δ2433_1661::1661	Derivative of ΔMSMEG_2433 carrying an unmarked, in-frame deletion in *M. smegmatis* MSMEG_2433 (870 bp internal region excised) in addition to MSMEG_1661 (1158 bp internal region excised). Complementation vector pMV306H_1661 carrying the entire MSMEG_1661 gene and 150bp upstream region inserted into the bacterial *attB* site.	This study

Δtriple::1661	Derivative of Δ2433_2432 carrying an unmarked, in-frame deletion of MSMEG_1661 (786 bp internal region excised). Complementation vector pMV306H_1661 carrying the entire MSMEG_1661 gene and 150bp upstream region inserted into the bacterial *attB* site.	This study
**Plasmids**		
p2NIL	*E. coli* cloning vector, Kan^r	(2)
pGOAL19	Plasmid carrying *lacZ-sacB-Hyg*^{r} markers as a *PacI* cassette; Hyg^r	(2)
p2Δ1661	Knockout vector for creating ΔMSMEG_1661 mutant. The 1.2 kbp upstream region including 18 bp of the 5' sequence of MSMEG_2433 was PCR-amplified to incorporate *Hin*DIII and *Nde*I restriction sites. The 1.2 kbp downstream was similarly obtained but incorporated at *Nde*I and *Acc*65I restriction sites. The fragments were cloned into p2NIL and the *PacI* cassette from pGOAL19 was inserted; Kan^r Hyg^r.	(26)
p2Δ2432	Knockout vector for creating ΔMSMEG_2432 mutant. The 1.2 kbp upstream region including 18 bp of the 5' sequence of MSMEG_2432 was PCR-amplified to incorporate *Hin*DIII and *Nde*I restriction sites. The 1.2 kbp downstream was similarly obtained but incorporated at *Nde*I and *Acc*65I restriction sites. The fragments were cloned into p2NIL and the *PacI* cassette from pGOAL19 was inserted; Kan^rHyg^r.	(26)
p2Δ2433	Knockout vector for creating ΔMSMEG_2433 mutant. The 1.2 kbp upstream region including 18 bp of the 5' sequence of MSMEG_2433 was PCR-amplified to incorporate *Hin*DIII and *Nde*I restriction sites. The 1.2 kbp downstream was similarly obtained but incorporated at *Nde*I and *Acc*65I restriction sites. The fragments were cloned into p2NIL and the *PacI* cassette from pGOAL19 was inserted; Kan^rHyg^r.	(26)

p2Δ2432-2433	Knockout vector for creating the simultaneous deletion of MSMEG_2432 and MSMEG_2433. The 1.2 kbp upstream region including 18 bp of the 5’ sequence of MSMEG_2432 and 1.2 kbp downstream region of MSEG_2433 was similarly obtained and compatible ends enabled direct ligation after restriction enzyme digestion. The fragments were cloned into p2NIL and the *PacI* cassette from pGOAL19 was inserted; $Kan^{r}Hyg^{r}$.	This study
pMV1661	Complementation vector containing full-length MSMEG_1661 and a small upstream region (150 bp) containing the native promoter region	This study

References

1. Snapper, S.B., et al., *Isolation and characterization of efficient plasmid transformation mutants of Mycobacterium smegmatis.* Mol Microbiol, 1990. **4**(11): p. 1911-9.
2. Parish, T. and N.G. Stoker, *Use of a flexible cassette method to generate a double unmarked Mycobacterium tuberculosis tlyA plcABC mutant by gene replacement.* Microbiology, 2000. **146 (Pt 8)**: p. 1969-75.
26. Ealand CS, Asmal R, Mashigo L, Campbell L, Kana BD. Characterization of putative DD-carboxypeptidase-encoding genes in *Mycobacterium smegmatis*. Sci Rep. 2019;9(1):5194; doi: 10.1038/s41598-019-41001-x.

Supplementary Figures

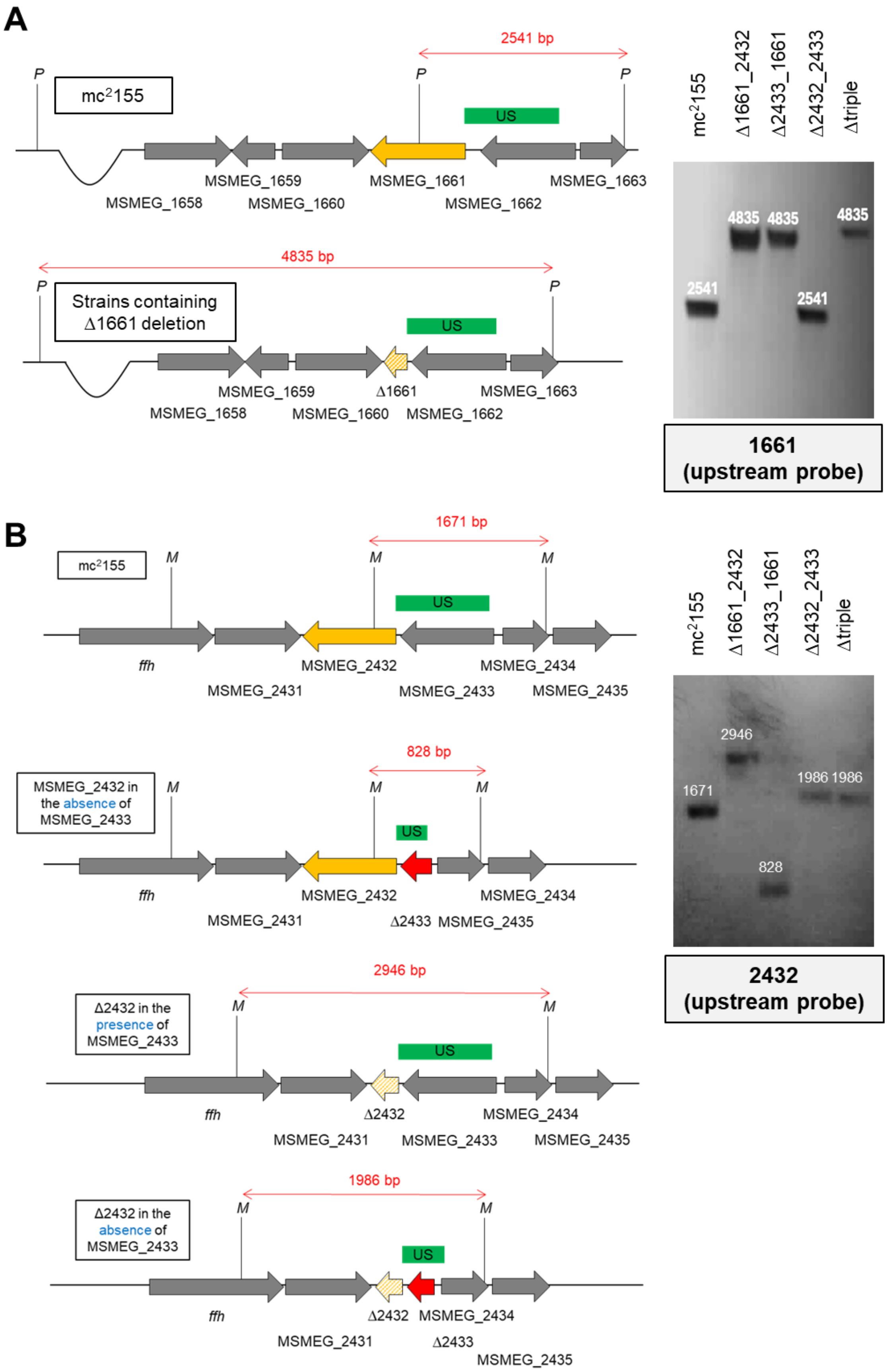

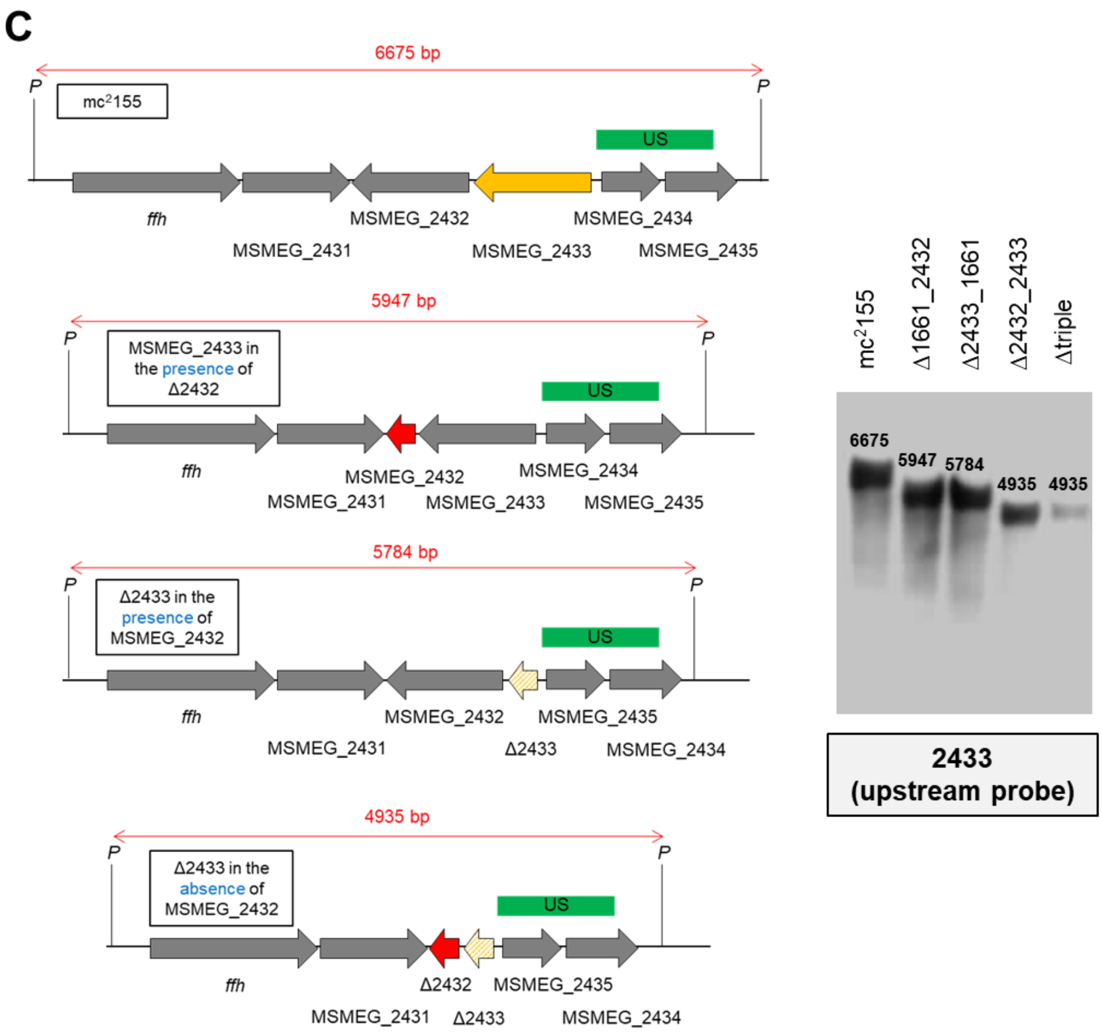

Figure S1. Southern hybridization to genotype all combinatorial DD-CPAse deletion mutants in *Mycobacterium smegmatis* mc^{2}155. (A) Genomic map and organization of wild-type (mc^{2}155) and mutant strains lacking MSMEG_1661. Southern blot in *PstI*-digested gDNA from mc^{2}155, Δ1661_2432, Δ2433_1661, Δ2432_2433 and Δ1661_2432_2433 (triple). Expected fragment sizes are shown in red and marked accordingly on the blot. (B) Genomic map and organization of wild-type (mc^{2}155) and mutant strains lacking MSMEG_2432. DNA was digested with *MluI* and loaded as described above. (C) Genomic map and organization of wild-type (mc^{2}155) and mutant strains lacking MSMEG_2433. DNA was digested with *PstI* and loaded as described above. In all cases, DIG-labelled probes were used to detect regions of interest indicated by green boxes.

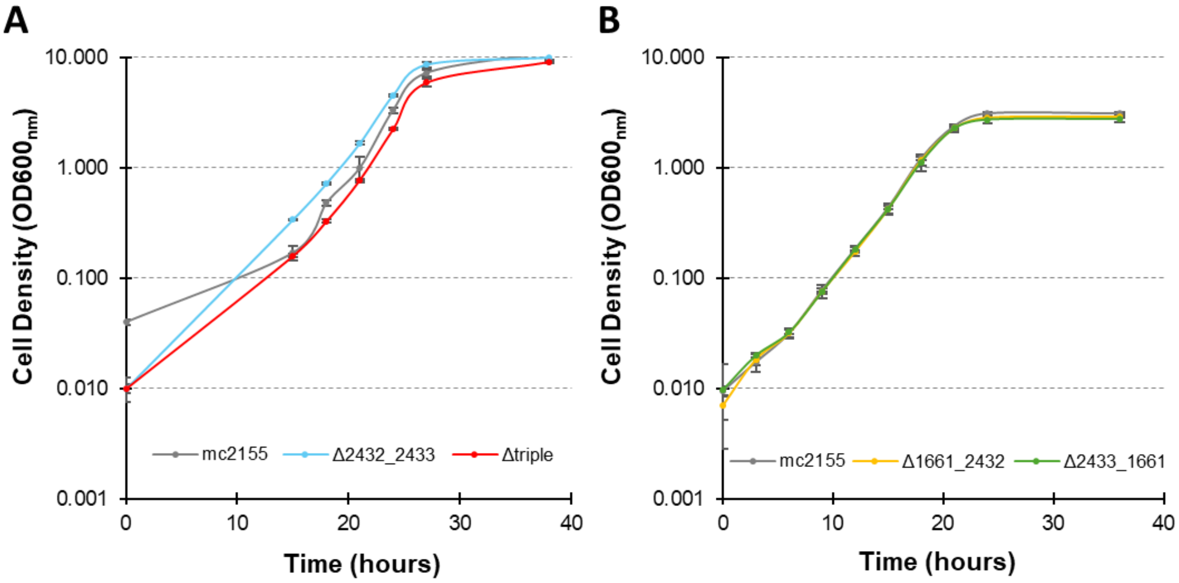

Figure S2. Growth kinetics of *M. smegmatis* strains lacking various combinations of DD-CPase homologues. (A) Growth of mc^{2}155, Δ2432_2433 and Δtriple in 7H9 media. **(B)** Growth of mc^{2}155, Δ1661_2432 and Δ2433_1661 in 7H9 media. In each case, data presented is an average of three independent experiments with error bars representing standard error of the mean. Growth curves were conducted on different days corresponding to when strains were initially constructed. The wildtype strain (mc^{2}155) served as a calibrator in both sets of experiments and confirmed that growth and viability was not negatively affected following DD-CPase gene deletions.

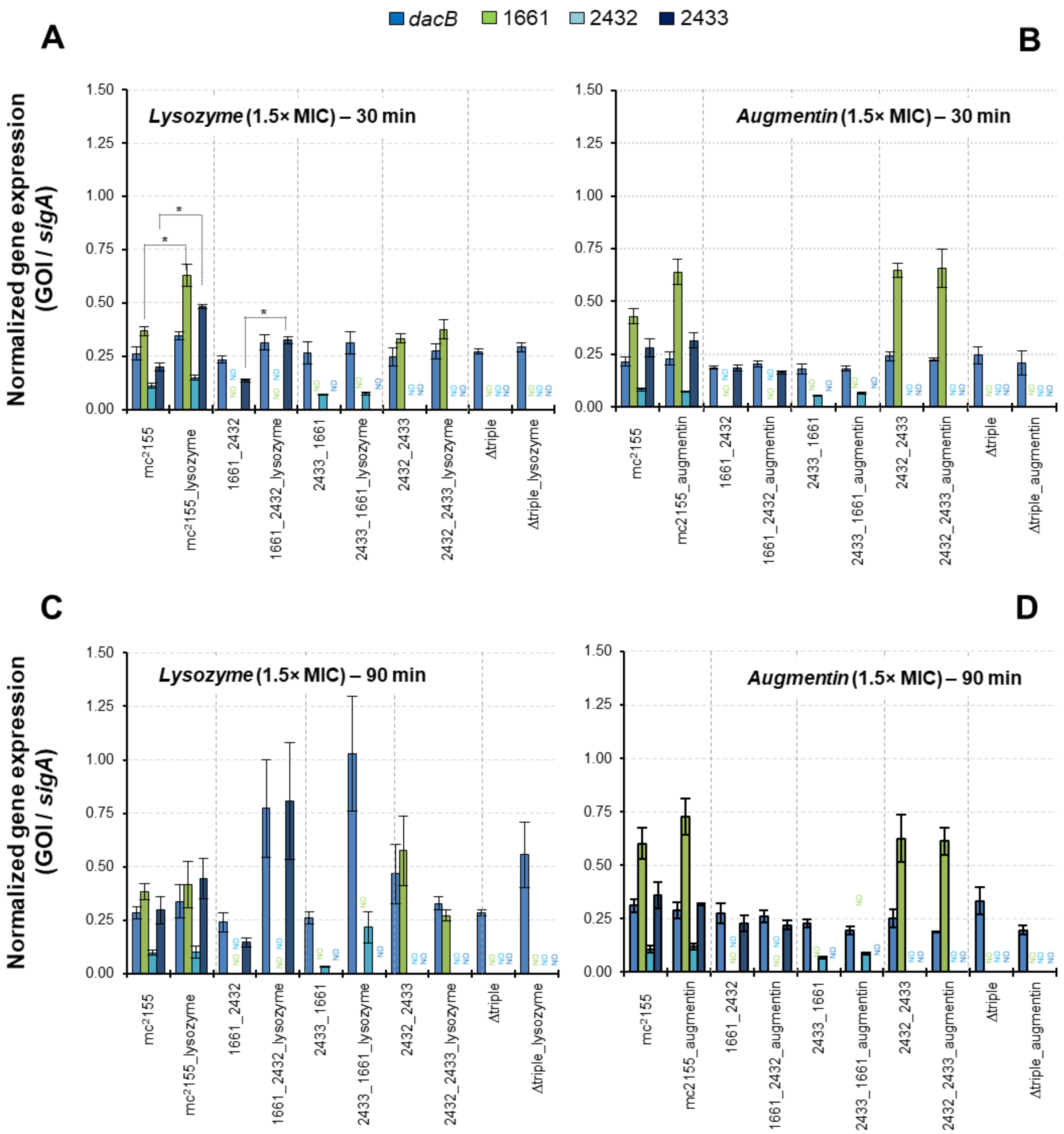

Figure S3. Determination of compensatory expression by remaining DD-CPase homologues in the respective combinatorial deletion mutant strains. (A) The expression of DD-CPase genes in strains exposed to lysozyme (1.5^{x} MIC at 58.5 µg/ml) for 30 min. Cultures lacking lysozyme served as the negative control. **(B)** The expression of DD-CPase genes in strains exposed to Augmentin (1.5^{x} MIC at 12 µg/ml amoxicillin + 4 µg/ml clavulanate) for 30 min. No drug exposure served as the negative control. **(C)** The expression of DD-CPase genes in strains exposed to lysozyme (1.5^{x} MIC at 58.5 µg/ml) for 90 min. Cultures lacking lysozyme served as the negative control. **(D)** The expression of DD-CPase genes in strains exposed to Augmentin (1.5^{x} MIC 12 µg/ml amoxicillin + 4 µg/ml clavulanate) for 90 min. No drug exposure served as the negative control. Under all experimental conditions, data are representative of three independent biological repeats. Error bars depict the standard error of the mean. *P*<0.05 (considered statistically significant) was determined using t-tests comparing the means of

transcript levels in the presence or absence of lysozyme or Augmentin (two-sample assuming unequal variances). Only statistically significant differences are shown with an *.

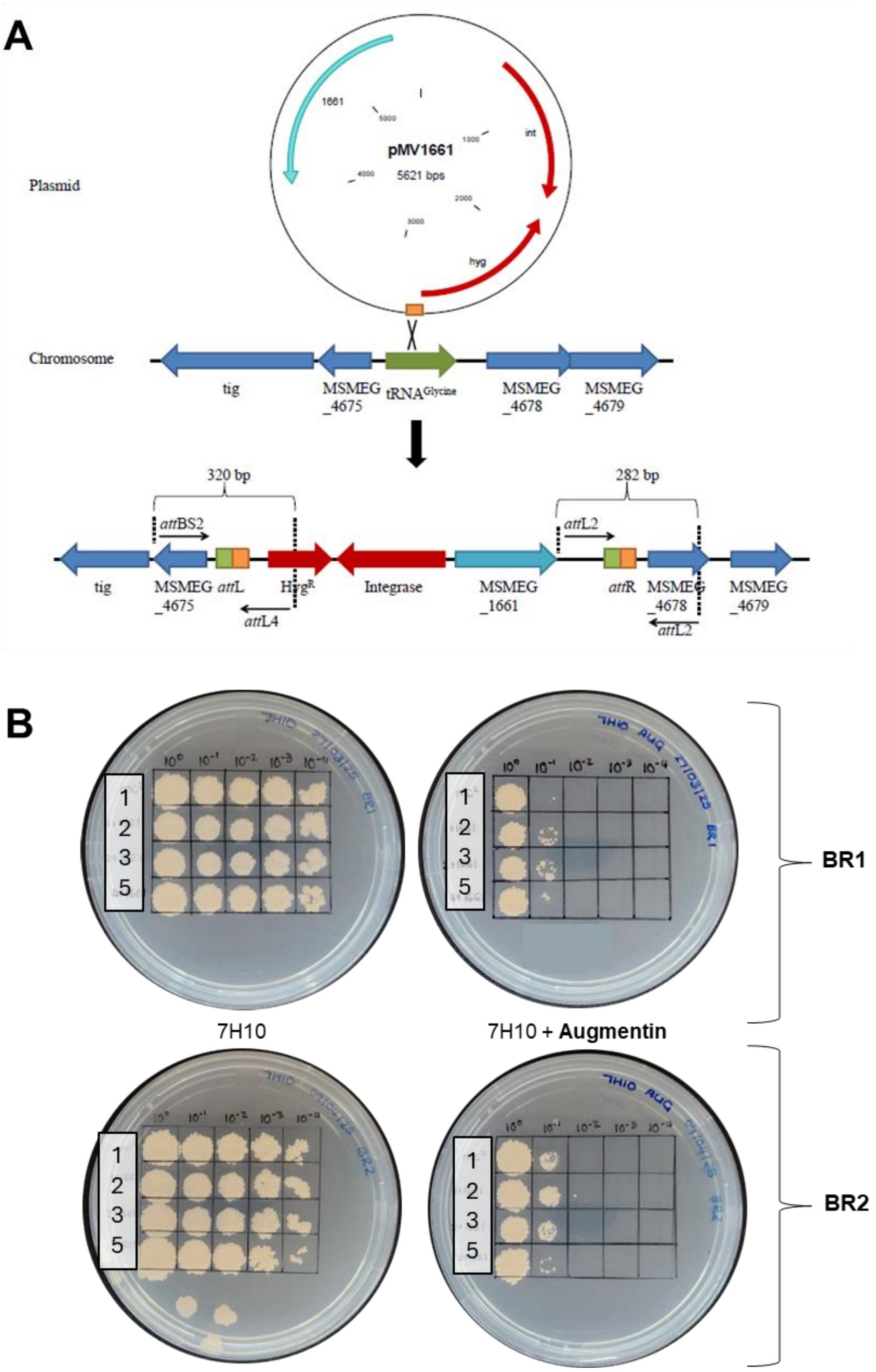

Figure S4. Schematic representation of integration of pMV1661 into the chromosome of deletion mutants and complementation effect in the presence of Augmentin. (A) The pMV1661 vector uses an L5 based integration system. The attachment site (orange block) integrates at the tRNAGly locus on the chromosome (green arrow), which results in the incorporation of the tRNAGly locus on either the left or right side of the plasmid, *attL* and *attR*. Primers used to confirm the site specific integration of pMV1661 and expected amplicon sizes are shown and integration was confirmed. **(B)** The wildtype strain (mc^{2}155) and only mutant strains simultaneously lacking were grown to exponential phase. Each

(1=mc^{2}155; 2= ∆1661_2432::1661; 3=∆2433_1661::1661; and 5=∆triple::1661) was diluted 10-fold and 5 µl of each dilution was spotted onto media. Conditions included 7H10 only (serving as a control) or Augmentin (3.91 µg/ml amoxicillin + 4 µg/ml clavulanate). Plates were then incubated at 37°C for 3-4 days followed by scoring. Images are representative of two independent biological repeats (BR1-2).

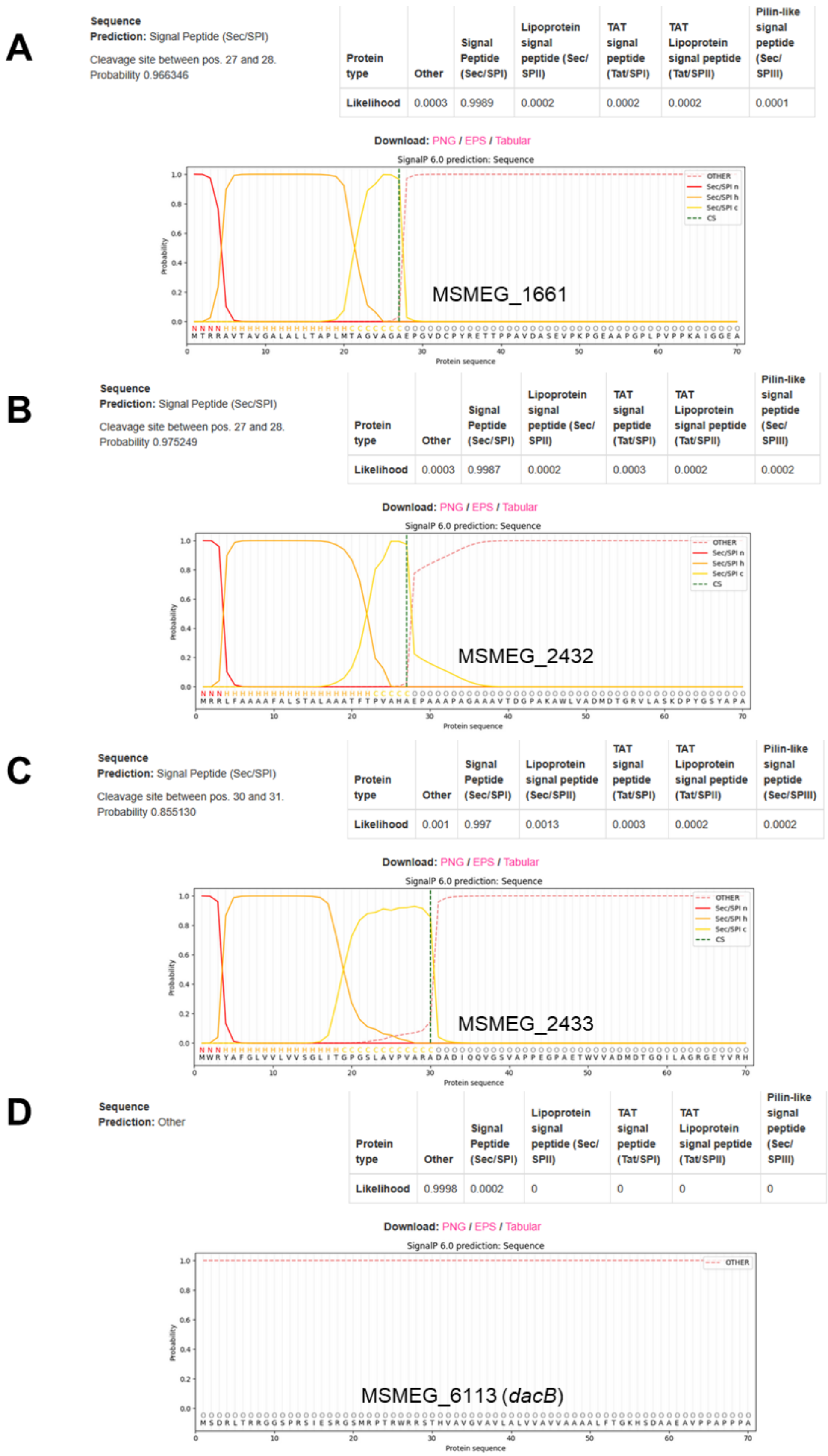

Figure S5. Signal sequences present in *M. smegmatis* DD-CPase proteins. Protein sequences were obtained from https://mycobrowser.epfl.ch/ and analysed using (https://services.healthtech.dtu.dk/services/SignalP-6.0/). Default parameters (Organism='other'; Output format='long output'; Model mode='Fast').

A

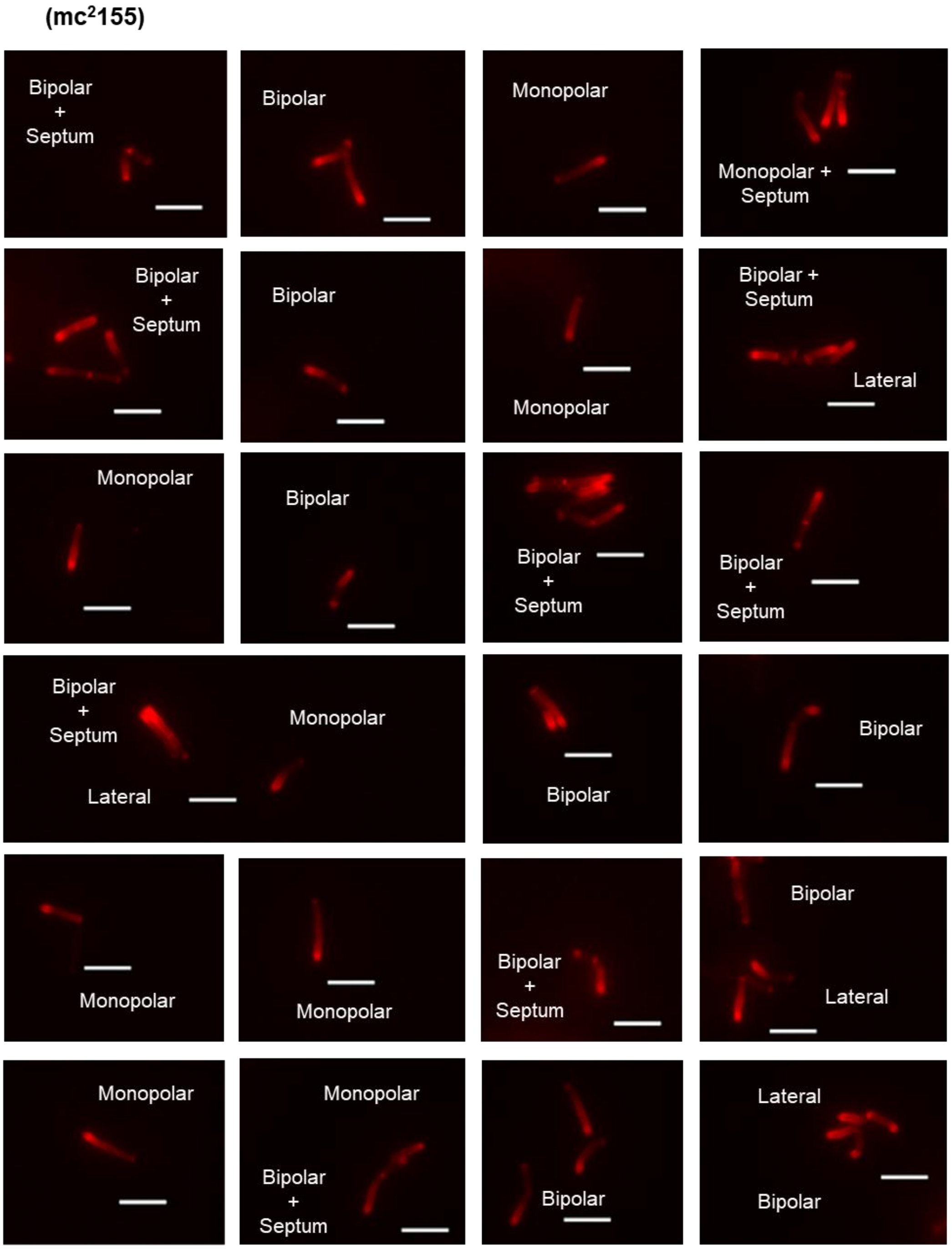
(mc2155)
Bipolar + Septum
Bipolar
Monopolar
Monopolar + Septum
Bipolar + Septum
Bipolar
Monopolar
Bipolar + Septum
Lateral
Monopolar
Bipolar
Bipolar + Septum
Bipolar + Septum
Bipolar + Septum
Lateral
Monopolar
Bipolar
Bipolar
Monopolar
Monopolar
Bipolar + Septum
Bipolar
Lateral
Monopolar
Monopolar
Bipolar + Septum
Bipolar
Lateral
Bipolar

B

(Δ1661_2432)

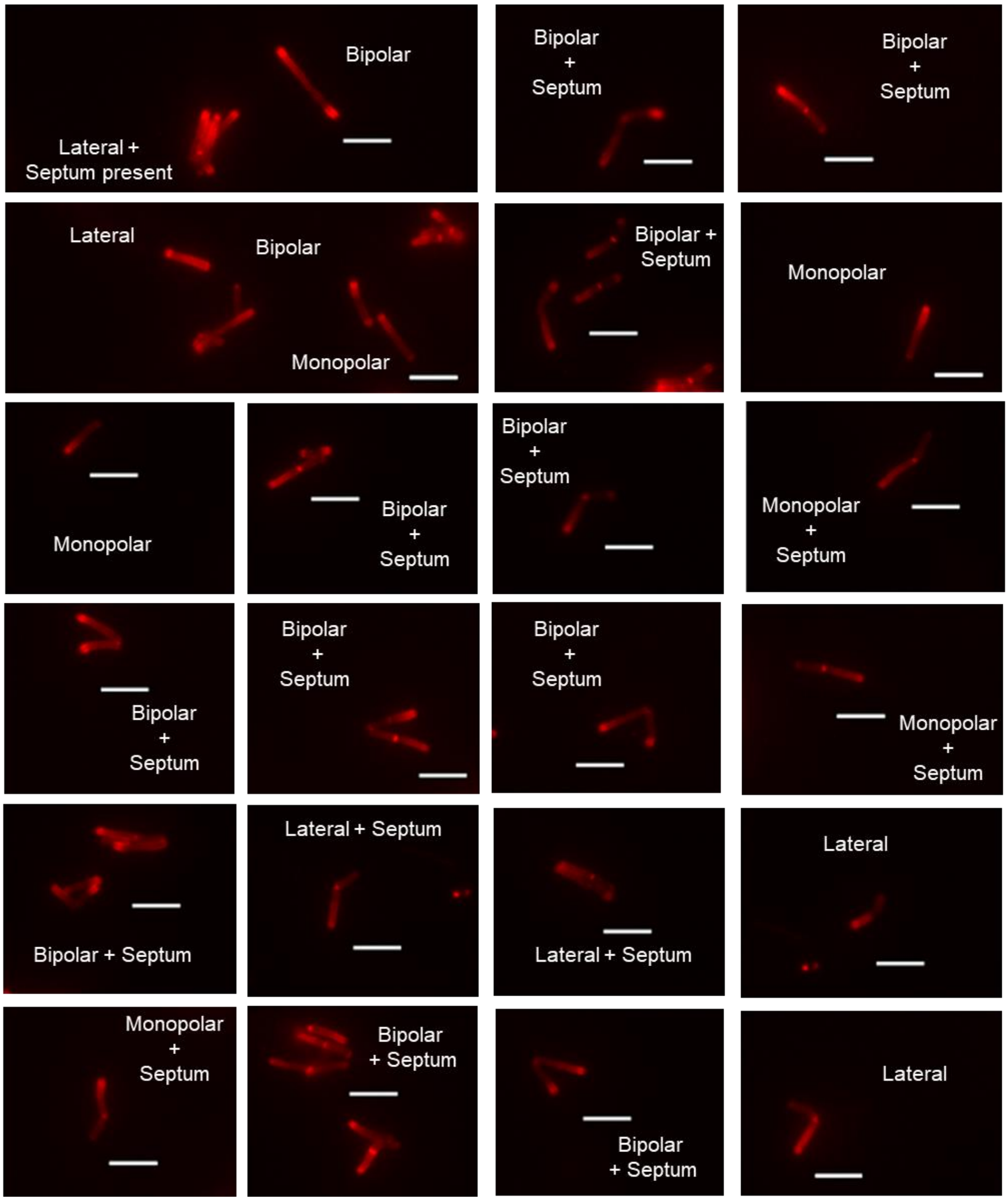

Bipolar
Lateral + Septum present
Bipolar + Septum
Bipolar + Septum
Lateral
Bipolar
Monopolar
Bipolar + Septum
Monopolar
Monopolar
Bipolar + Septum
Bipolar + Septum
Monopolar + Septum
Bipolar + Septum
Bipolar + Septum
Bipolar + Septum
Monopolar + Septum
Bipolar + Septum
Lateral + Septum
Lateral + Septum
Lateral
Monopolar + Septum
Bipolar + Septum
Bipolar + Septum
Lateral

C

(Δ2433_1661)

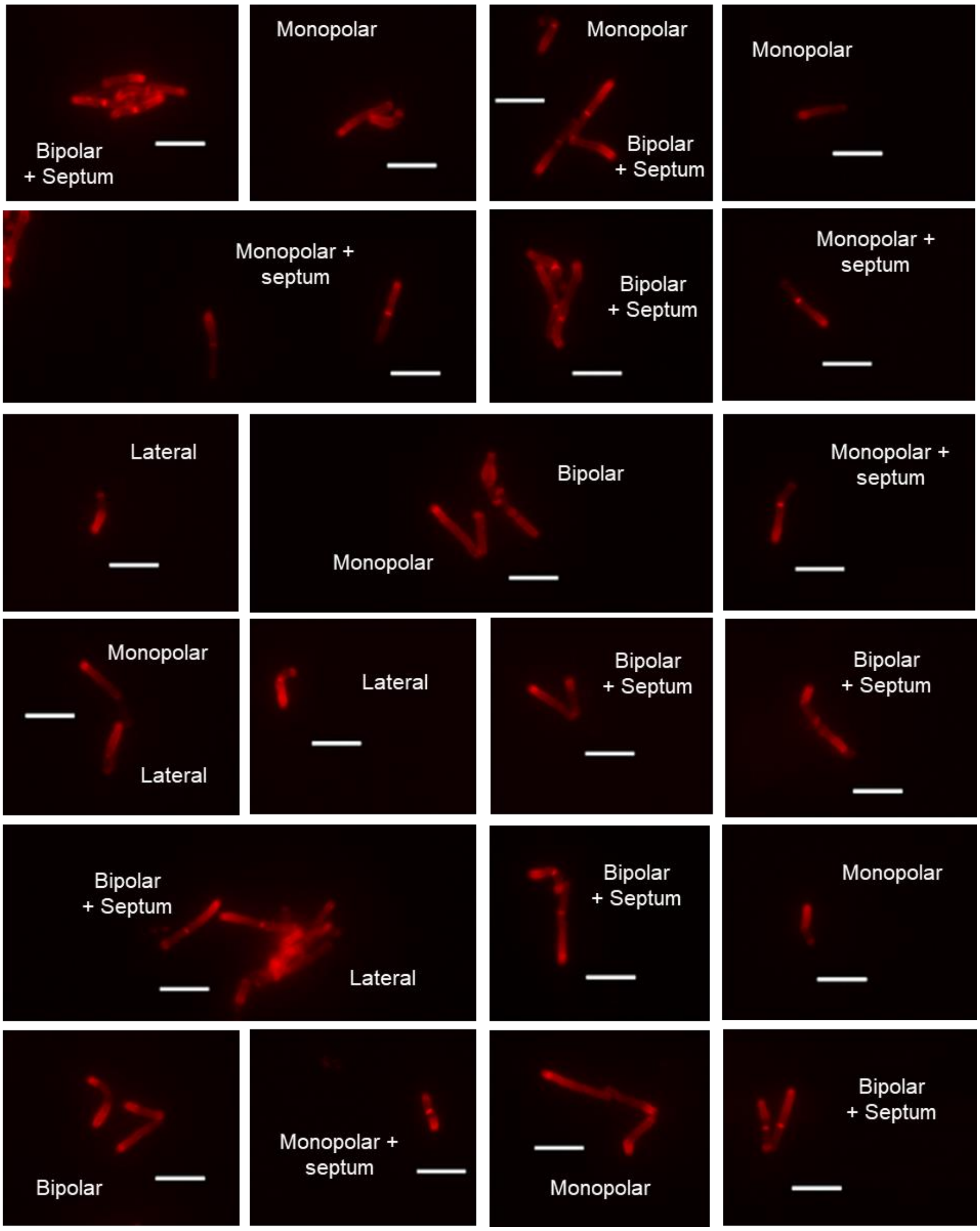
Bipolar
+ Septum
Monopolar
Monopolar
Bipolar
+ Septum
Monopolar
Monopolar +
septum
Bipolar
+ Septum
Monopolar +
septum
Lateral
Bipolar
Monopolar
Monopolar +
septum
Monopolar
Lateral
Lateral
Bipolar
+ Septum
Bipolar
+ Septum
Bipolar
+ Septum
Lateral
Bipolar
+ Septum
Monopolar
Bipolar
Monopolar +
septum
Monopolar
Bipolar
+ Septum

D

(Δ2432_2433)

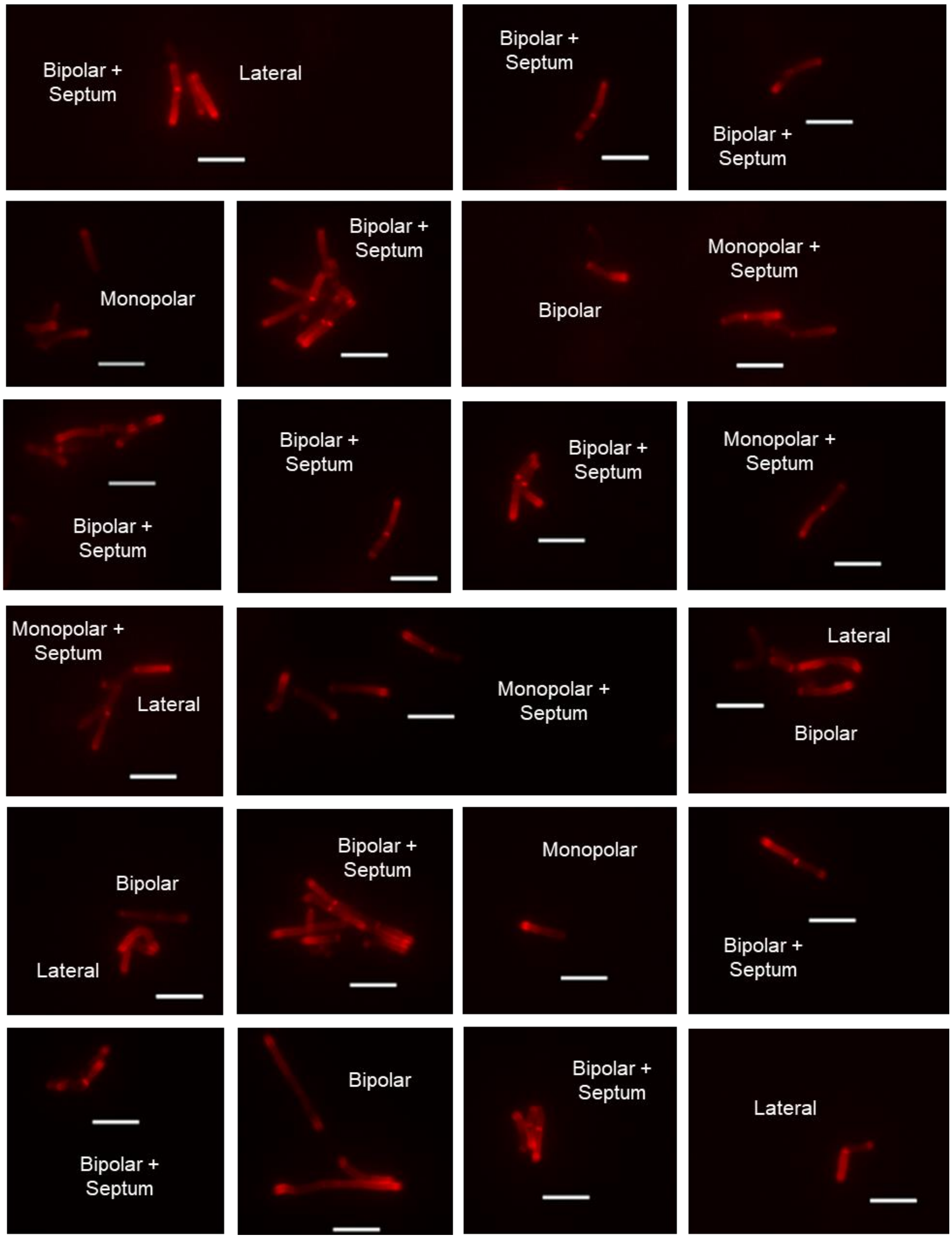

Bipolar + Septum
Lateral
Bipolar + Septum
Bipolar + Septum
Monopolar
Bipolar + Septum
Monopolar + Septum
Bipolar
Bipolar + Septum
Bipolar + Septum
Bipolar + Septum
Monopolar + Septum
Monopolar + Septum
Lateral
Monopolar + Septum
Lateral
Bipolar
Bipolar
Lateral
Bipolar + Septum
Monopolar
Bipolar + Septum
Bipolar + Septum
Bipolar
Bipolar + Septum
Lateral

E

(Δtriple)

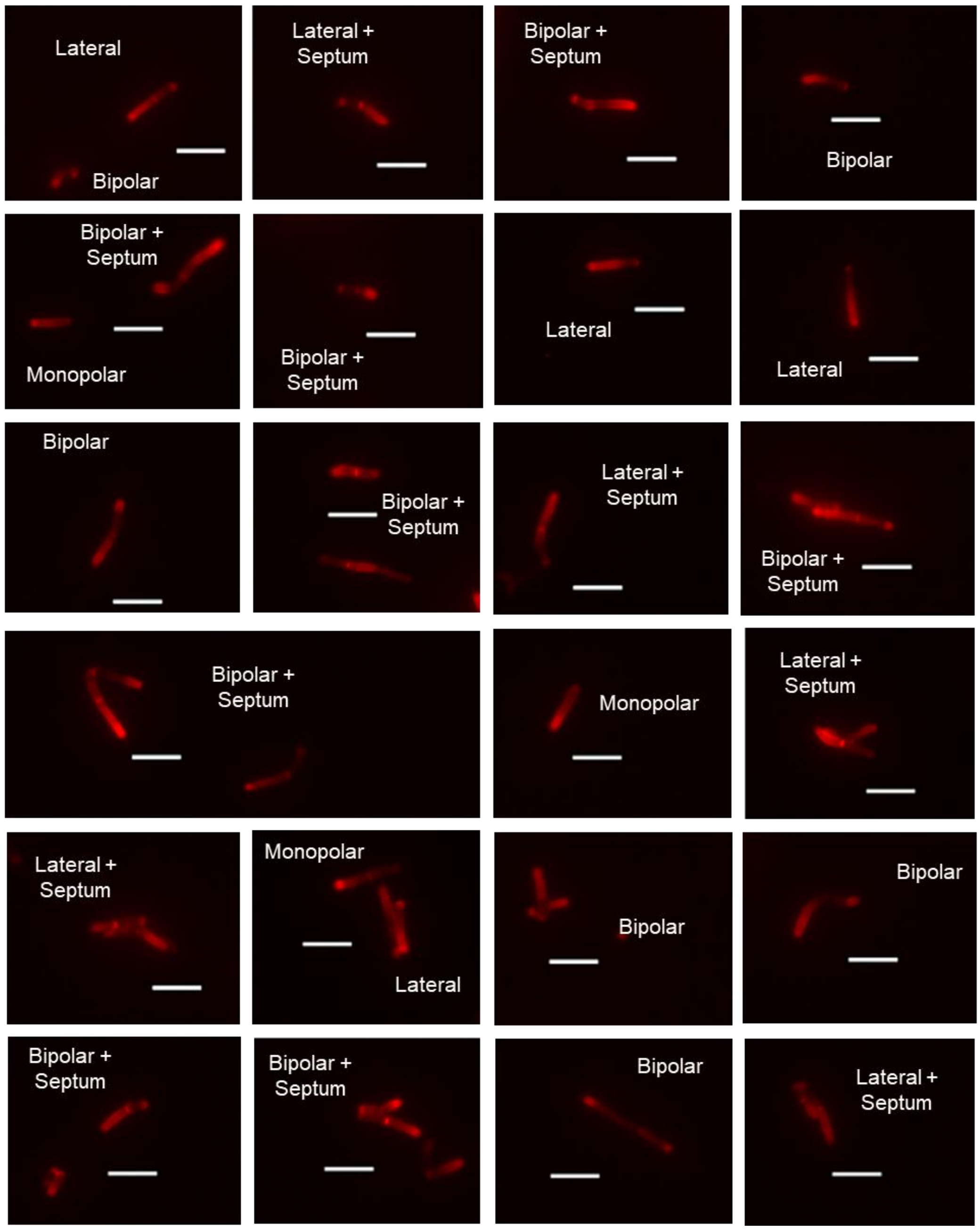

Figure S6. Representative images of mutant strains treated with lysozyme followed by staining with fluorescent, D-amino acid mono-peptide dye (TADA, 5 µg/ml). (A – E) represent the wild-type (mc^{2}155), Δ1661_2432, Δ2433_1661, Δ2432_2433 and Δtriple, respectively. Following lysozyme treatment, bacteria were stained with TADA (excitation / emission wavelengths = 554 / 580 nm, respectively) shown here. The scale bar is equivalent to 5 µm. Staining patters were characterized as 'bipolar', 'monopolar', 'lateral' with/without a 'septum present'. Three independent biological repeats were performed for each strain (n = 100 for each repeat).
